# Molecular mechanism of thermosensory function of human heat shock transcription factor Hsf1

**DOI:** 10.7554/eLife.11576

**Published:** 2016-01-19

**Authors:** Nikolai Hentze, Laura Le Breton, Jan Wiesner, Georg Kempf, Matthias P Mayer

**Affiliations:** Zentrum für Molekulare Biologie der Universität Heidelberg, Heidelberg, Germany; University of Cambridge, United Kingdom

**Keywords:** conformational dynamics, heat shock response, heat shock transcription factor, temperature response, thermosensor, hydrogen exchange mass spectrometry, None

## Abstract

The heat shock response is a universal homeostatic cell autonomous reaction of organisms to cope with adverse environmental conditions. In mammalian cells, this response is mediated by the heat shock transcription factor Hsf1, which is monomeric in unstressed cells and upon activation trimerizes, and binds to promoters of heat shock genes. To understand the basic principle of Hsf1 activation we analyzed temperature-induced alterations in the conformational dynamics of Hsf1 by hydrogen exchange mass spectrometry. We found a temperature-dependent unfolding of Hsf1 in the regulatory region happening concomitant to tighter packing in the trimerization region. The transition to the active DNA binding-competent state occurred highly cooperative and was concentration dependent. Surprisingly, Hsp90, known to inhibit Hsf1 activation, lowered the midpoint temperature of trimerization and reduced cooperativity of the process thus widening the response window. Based on our data we propose a kinetic model of Hsf1 trimerization.

**DOI:** http://dx.doi.org/10.7554/eLife.11576.001

## eLife digest

Cells cope with excessive heat, toxic compounds and other adverse environmental conditions by triggering an internal repair process called the heat shock response. In mammalian cells, a protein called Hsf1 is activated by stress and regulates the activity of a large set of target genes. These genes code for proteins that help the cell cope with the effects of stress, for example, by repairing or breaking down damaged proteins. Under normal conditions, Hsf1 exists as a single molecule, but when it is activated, three molecules come together to make a complex called a trimer that is able to bind to DNA and activate the target genes.

Proteins are made of long chains that then fold into specific three-dimensional shapes. It is not known how Hsf1 is kept in an inactive state in healthy, unstressed cells. One possibility is that the protein folds into a three-dimensional shape that prevents it from being activated. Alternatively, Hsf1 may be bound to other proteins called chaperones that move away when the cell is under stress because they are needed to help the damaged proteins refold into their own three-dimensional shapes.

Hentze et al. used a variety of biochemical techniques to study the human Hsf1 protein. The experiments showed that there are two regions of the Hsf1 protein that changed shape dramatically when the temperature increased. A region that regulates the activity of Hsf1 unfolded, while a region involved in making the trimer became more stable. Detailed analysis showed that once the regulatory region unfolded, the protein was able to interact with other Hsf1 units to make the trimer. Therefore, Hsf1 can directly sense and respond to changes in temperature without the aid of any chaperone proteins.

Further experiments showed that the formation of Hsf1 trimers and the ability of these trimers to bind to DNA depend upon both the temperature and the amount of Hsf1 present. In addition, a chaperone protein called Hsp90 – which is known to be able to interact with Hsf1 – influenced how Hsf1 responded to changes in temperature. Hentze et al. also present a model for the activation of Hsf1 that allows for flexibility in the response of Hsf1 to changes in temperature. Previous studies have shown that Hsf1 is chemically modified during stress and also while the cell recovers from stressful conditions. Therefore, the next challenge will be to find out how these modifications influence the way in which Hsf1 responds to stress.

**DOI:** http://dx.doi.org/10.7554/eLife.11576.002

## Introduction

To cope with changes in physical and chemical properties of the environment as well as with physiological and pathophysiological conditions which cause protein misfolding, organisms mount a homeostatic transcriptional program, the so-called heat shock response (Jolly and Morimoto, 2000). In all eukaryotic cells, heat shock transcription factor (HSF) 1 is the master regulator of this response and alters transcription of a large number of genes, some of which encode chaperones and proteases (Anckar and Sistonen, 2011). Although this response is essentially cell autonomous, systemic modulation of this response has been observed in metazoa (Morimoto, 2008; Prahlad et al., 2008; Prahlad and Morimoto, 2011).

**Figure 1.**
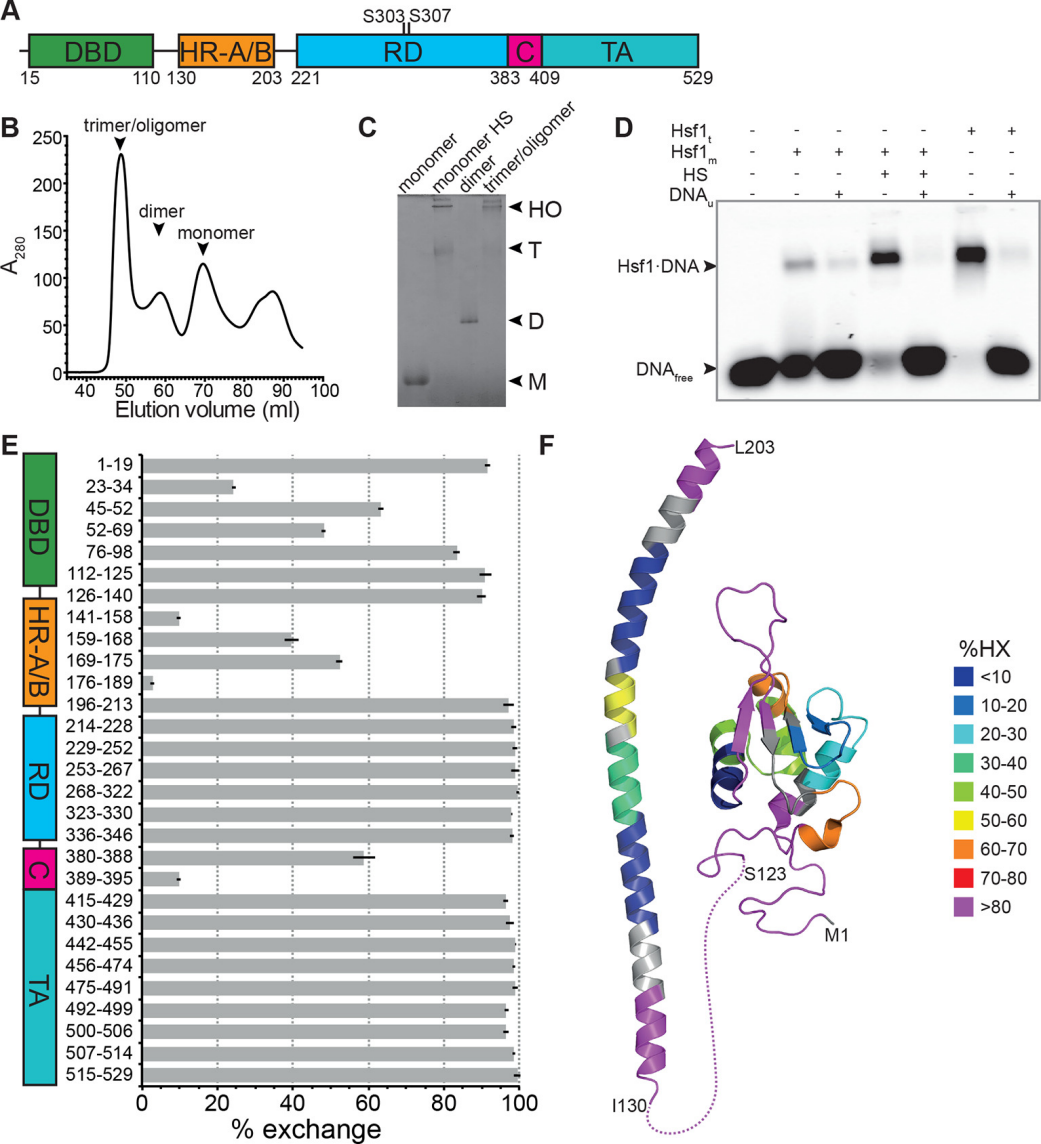
Recombinant purified human Hsf1 is largely monomeric and trimerizes and acquires DNA binding competence upon heat shock. (**A**) Domain organization of human Hsf1 [modified from Anckar and Sistonen (2011)]. (**B**) Size exclusion chromatography separates recombinant human Hsf1 in monomer, dimer and trimer/oligomer as indicated. (**C**) Blue native gel of the three peak indicated in panel B (monomer and dimer), monomeric Hsf1 after 10 min heat shock at 42°C (monomer HS); and trimeric/oligomeric Hsf1 purified under denaturing conditions and refolded into a DNA binding competent state (M, monomer; D, dimer; T, trimer; HO, higher order oligomers). (**D**) Electrophoretic mobility shift assay (EMSA). Monomeric Hsf1 (Hsf1_m_), monomeric Hsf1 treated for 10 min at 42°C (HS), or trimeric Hsf1 (Hsf1_t_) were incubated with fluorescent labeled HSE-DNA minus or plus unlabeled HSE-DNA and separated on a native agarose gel. Lane 1, HSE-DNA in the absence of protein. (**E**) Amide hydrogen exchange of monomeric Hsf1 after 30 s at 20°C in D_2_O buffer. Exchange was correct for back exchange using a fully deuterated Hsf1 preparation. Error bars are the standard error of mean (SEM) of three independent experiments. (**F**) Cartoon representation of the DNA binding (PDB ID 2LDU) and trimerization domains of human Hsf1 colored according to deuteron incorporation as indicated. Gray, no sequence coverage. The trimerization domain is a homology model of the HR-A/B region (residues 130–203) of human HSF1 on the structure of *Chaetomium thermophilum* Skn7 [PDB ID 5D5Z, (Neudegger et al., 2016) using I-TASSER (Zhang, 2008; Yang and Zhang, 2015; Yang et al., 2015; Roy et al., 2010)]. **DOI:** http://dx.doi.org/10.7554/eLife.11576.003

Metazoan Hsf1 consists of a N-terminal winged helix-turn-helix DNA binding domain (Harrison et al., 1994; Vuister et al., 1994), a hydrophobic shorter heptad repeat regions (HR-A/B) proposed to function as a leucine zipper coiled-coil trimerization domain (Clos et al., 1990; Rabindran et al., 1993), a regulatory domain, a second heptad repeat (HR-C) and a C-terminal transcription activation domain (Figure 1A) (Anckar and Sistonen, 2011; Voellmy, 2004). In unstressed cells metazoan Hsf1 is monomeric and supposed to be in complex with molecular chaperones, including Hsp70, Hsp90 and TRiC/CCT (Shi et al., 1998; Zou et al., 1998; Neef et al., 2014). At physiological concentrations monomeric Hsf1 does not bind appreciably to heat shock elements (nGAAn). In the activated state Hsf1 forms trimers or higher order oligomers and binds to its response elements in heat shock gene promoters (Clos et al., 1990; Rabindran et al., 1993). Currently, two models are discussed for the heat-induced activation of Hsf1: (1) Based on the observation that deletion or mutational alteration of HR-C leads to constitutive trimerization Wu and co-workers proposed that Hsf1 is a thermosensor itself and kept monomeric by intramolecular leucine zipper formation (Rabindran et al., 1993). However, activation of human Hsf1 in human and insect cells and *Xenopus* oocytes occurs at different temperatures, arguing against an Hsf1 intrinsic mechanism of heat shock activation (Baler et al., 1993; Clos et al., 1993). (2) Owing to the fact that the large variety of Hsf1-inducing signals have in common to cause protein misfolding and in analogy to the regulation of the heat shock response in *E. coli* (Guisbert et al., 2008), chaperones were proposed to prevent Hsf1 activation and to be titrated away from Hsf1 under stress conditions, resulting in heat shock response induction (Morimoto, 1998). Consistent with this hypothesis is the observation that inhibition of Hsp70, Hsp90 or TRiC/CCT or knock-down of their expression leads to the induction of the heat shock response (Powers and Workman, 2007; Powers et al., 2008; Neef et al., 2014; Whitesell et al., 2003; Lee et al., 2013; Abravaya et al., 1992; Zou et al., 1998).

Further regulation of Hsf1 is provided by posttranslational modifications, including phosphorylation, acetylation, sumoylation and oxidation of cysteines to disulfide bridges (Hietakangas et al., 2003; 2006; Sarge et al., 1993; Westerheide et al., 2009; Brunet Simioni et al., 2009; Zhong et al., 1998; Lu et al., 2008). The contribution of these modifications to the primary activating mechanism are still unclear (Budzyński et al., 2015).

To resolve the molecular mechanism of the temperature-induced activation of Hsf1 we analyzed the conformational dynamics of purified monomeric human Hsf1 pretreated at different temperatures using hydrogen-^1^H/^2^H-exchange (HX) mass spectrometry (MS). We found temperature-dependent unfolding of HR-C and concomitant stabilization of HR-A/B, demonstrating that isolated Hsf1 acts as temperature sensor. At short incubation times the temperature response curve exhibits high cooperativity with a transition midpoint of 36°C. Using fluorescence anisotropy we demonstrate that the acquisition of DNA-binding competency depends on temperature and concentration of Hsf1. Phosphomimetic Hsf1 variants corresponding to phosphorylation at two serine residues previously shown to negatively affect Hsf1 activation did not have an increased temperature transition midpoint. Hsp90 known to negatively regulate Hsf1-mediated transcription decreased the slope of the temperature response curve, thereby lowering the transition midpoint and widening the response window. Our data suggest a kinetic model of Hsf1 trimerization.

## Results

Recombinant human Hsf1 was purified out of *E. coli* by affinity chromatography and size-exclusion chromatography, resulting in mostly monomeric species in the final fraction (Figure 1B and C). Upon incubation at 42°C, Hsf1 formed trimers and higher-order oligomers, as verified by blue native gel electrophoresis consistent with published data (Clos et al., 1990), and acquired DNA-binding competence as shown by electrophoretic mobility shift assays (Figure 1C and D). The conformational dynamics of Hsf1 was investigated by HX-MS as described previously (Rist et al., 2006; Graf et al., 2009). Monomeric Hsf1 was incubated for 30 s in D_2_O at 20°C, subsequently mixed with ice-cold, low-pH quench buffer to slow down back exchange, and analyzed on our HPLC-mass spectrometry setup including a column with immobilized pepsin for online digestion. As shown in Figure 1E, monomeric Hsf1 is highly dynamic with only few regions exhibiting significant protection from HX, including parts of the DNA binding domain and the trimerization domain (HR-A/B). Out of the C-terminal half of the protein, containing the regulatory region, HR-C and the transactivation domain, only the HR-C region showed significant protection at 20°C consistent with an earlier study showing the C-terminal half of Hsf1 largely unfolded (Pattaramanon et al., 2007). Figure 1F shows a heat map of the DNA binding domain and the trimerization domain of Hsf1, the only parts for which structural information is available.

### Hsf1 is a thermosensor

**Figure 2.**
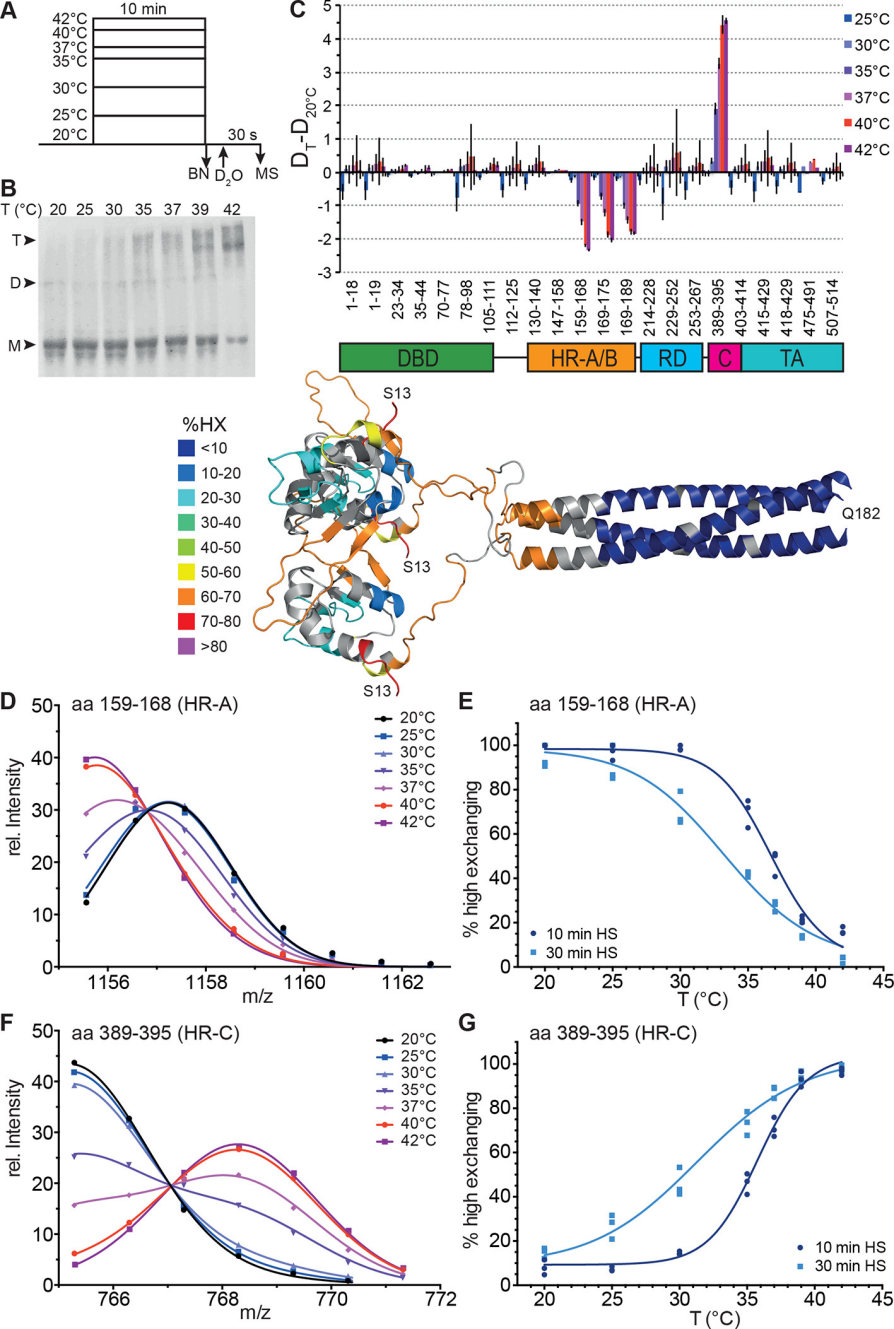
Human Hsf1 is a thermosensor. (**A**) Experimental design: monomeric human Hsf1 was pre-incubated at different temperature as indicated for 10 min or 30 min and then either analyzed by blue native polyacrylamide gel electrophoresis (BN) or diluted 20-fold into D_2_O-buffer at 20°C and incubated for 30 s. The reaction was quenched and the samples analyzed by HPLC-MS. (**B**) Analysis of quaternary structure of Hsf1 after pre-incubation at different temperatures for 10 min as indicated. Hsf1 was detected by immunoblotting with an Hsf1 specific antiserum. M, monomer; D, dimer; T, trimer. (**C**) Difference plot of deuteron incorporation into monomeric Hsf1 pre-incubated at the indicated temperature minus deuteron incorporation of Hsf1 pre-incubated at 20°C for peptic peptides as indicated. Error bars are SEM of three independent experiments. Cartoons underneath the X-axis indicate the domains of Hsf1 corresponding to the respective peptic peptides and a homology model of the trimerized human HSF1 (kindly provided by A. Bracher [Neudegger et al., 2016]), colored according to HX as indicated. First and last amino acid of the model are indicated. (**D, F**) Intensity distributions of the isotope clusters of peptide 1155.58^1+^ corresponding to amino acids 159–168 (**D**) and 765.30^1+^ corresponding to amino acids 389–395 (**F**) for different pre-incubation temperatures, as indicated. Curves are fits of the sum of two Gaussian peak functions to the data (see Figure 2—figure supplement 2). Representative plot of three independent experiments. (**E, G**) Fraction of high-exchanging species calculated, as described in Figure 2—figure supplement 2. Data points for three independent experiments with 10-min (dark blue) and 30-min (light blue) pre-incubation time at elevated temperatures are shown for peptide 159–168 (**E**) and 389–395 (**G**). The curve is a fit of a thermal unfolding equilibrium to the data. Data for additional peptides are shown in Figure 2—figure supplement 3. **DOI:** http://dx.doi.org/10.7554/eLife.11576.004

To elucidate temperature-induced changes in conformational dynamics, we pre-incubated monomeric Hsf1 at different temperatures for different time intervals followed by incubation at constant temperature in D_2_O (Figure 2A). As control, we analyzed the pre-treated Hsf1 by blue native polyacrylamide gel electrophoresis (Wittig et al., 2006) and observed a temperature-dependent increase in trimeric Hsf1 species (Figure 2B and Figure 2—figure supplement 1). The 10 min-pre-incubation of Hsf1 dramatically changed conformational dynamics of two regions in Hsf1 (Figure 2): temperature-dependent increase in HX is observed in HR-C, indicating heat-induced unfolding, and a concomitant decrease in HX is observed in HR-A/B, consistent with heat-induced trimerization. Close inspection of the spectra of the peptic fragments exhibiting temperature-induced changes in HX revealed bimodal distributions of the isotope clusters indicative of the coexistence of two populations of molecules with different exchange properties (Figure 2—figure supplement 2A and C, Figure 2—figure supplement 3). An equation for two Gaussian curves was fitted to the intensity-versus-m/z plots of the data (Figure 2D and F, and Figure 2—figure supplement 2B and D, Figure 2—figure supplement 3) and the equation parameters used to back calculate the contribution of each population to the peak intensities (see Figure 2—figure supplement 2). For the HR-A/B region the relative frequency of high exchanging population decreases with pre-incubation at increasing temperatures resulting in a sigmoidal temperature response curve (Figure 2E). For the HR-C region the opposite is observed: the frequency of high exchanging species increased with increasing temperatures (Figure 2G). These data clearly demonstrate that Hsf1 has intrinsic thermosensory properties. The midpoint of transition T_m_, the temperature at which 50% of the molecules are in the high exchanging conformation after 10 min, was identical for both regions equal to 36.15 ± 0.14°C.

**Figure 2—figure supplement 1.**
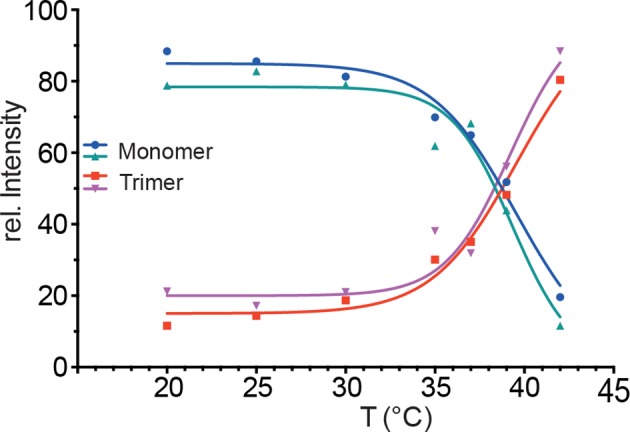
Temperature-induced transition of Hsf1 from the monomeric into the trimeric state as determined by blue native polyacrylamide gel electrophoresis. Quantification of immune blots with Hsf1-specific antisera of blue native gels of two independent experiments, one of which is shown in Figure 2B. Curves are fits of the thermal unfolding equation to the data, restricting the minimal value to 0 and the maximal value to 100. **DOI:** http://dx.doi.org/10.7554/eLife.11576.005

**Figure 2—figure supplement 2.**
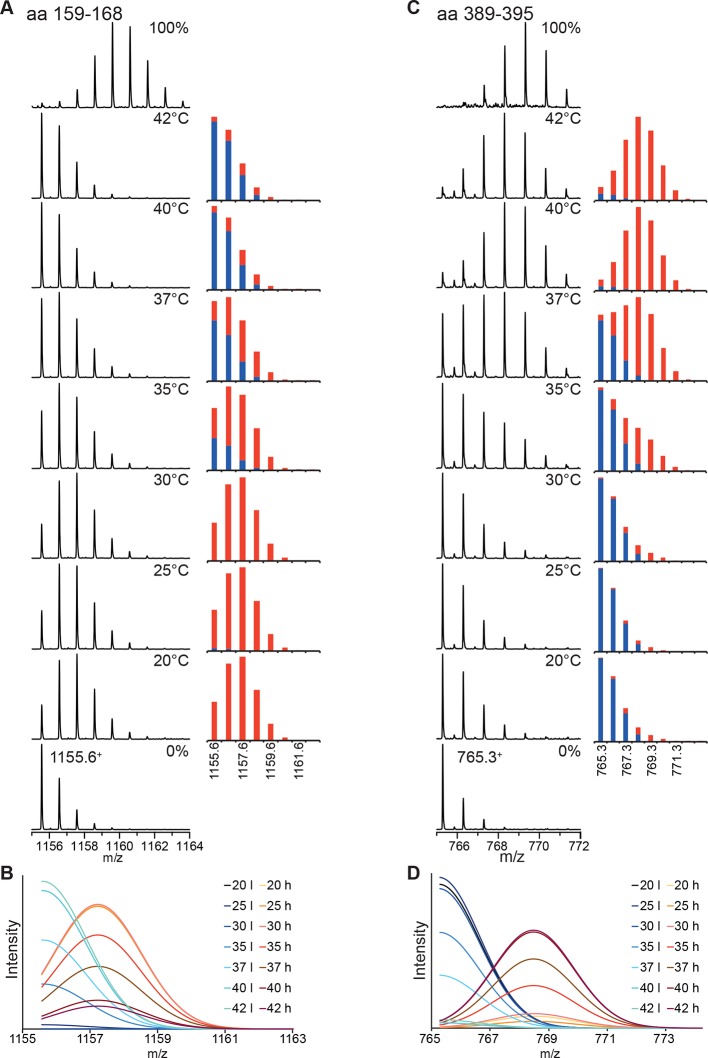
Analysis of the bimodal distributions of the isotope clusters detected by MS. (**A, C**) Original spectra of peptic peptide 1155.6^1+^ (amino acids 159–168; A left) and 765.3^1+^ (aa 389–395; **C**, left) and fractional peak intensities (**A**, right, **C**, right) for low (blue) and high (red) exchanges species calculated from the parameters of the fits of the sum of two Gaussian peaks to the data shown in Figure 2. Unexchanged and 100% control are shown at the bottom and top, respectively. (**B, D**) Individual Gaussian peaks for high (h) and low (l) exchanging species for the indicated temperatures the sum of which results in the fits shown in Figure 2. **DOI:** http://dx.doi.org/10.7554/eLife.11576.006

**Figure 2—figure supplement 3.**
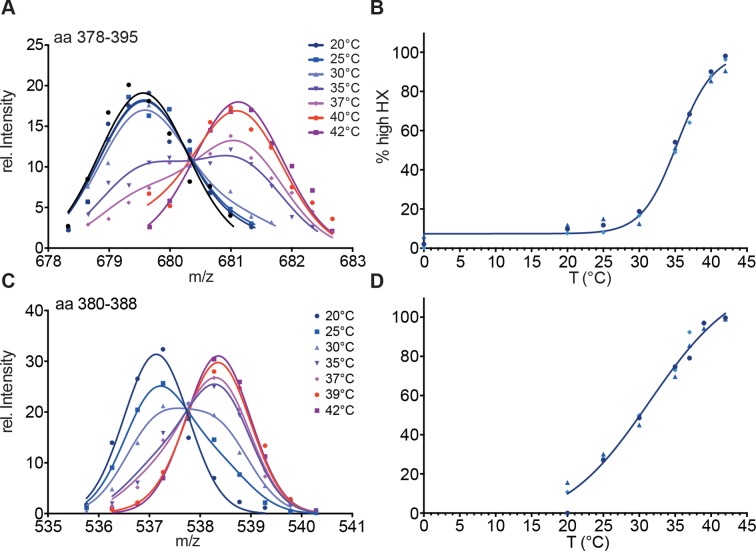
Hsf1 is a thermosensor. (**A**) Intensity distributions of the isotope clusters of the peptic peptide 678.3^3+^ corresponding to amino acids 378–395 of Hsf1 pre-incubated for 10 min at different temperatures as indicated. One representative plot of three independent experiments is shown. (**B**) Fraction of high-exchanging species calculated, as described in Figure 2—figure supplement 2. Data points for three independent experiments are shown in different shades of blue for peptide 378–395. The curve is a fit of a thermal unfolding equilibrium to the data. (**C**) Incubation for 30 min leads to shallower transition curves. Intensity distributions of the isotope clusters of peptic peptide 535.8^2+^ corresponding to amino acids 380–388 of Hsf1 pre-incubated for 30 min at different temperatures, as indicated. One representative plot of three independent experiments is shown. (**D**) Fraction of high exchanging species calculated, as described in Figure 2—figure supplement 2. Data points for three independent experiments are shown in different shades of blue for peptide 380–388. The curve is a fit of a thermal unfolding equilibrium to the data. **DOI:** http://dx.doi.org/10.7554/eLife.11576.007

**Figure 2—figure supplement 4.**
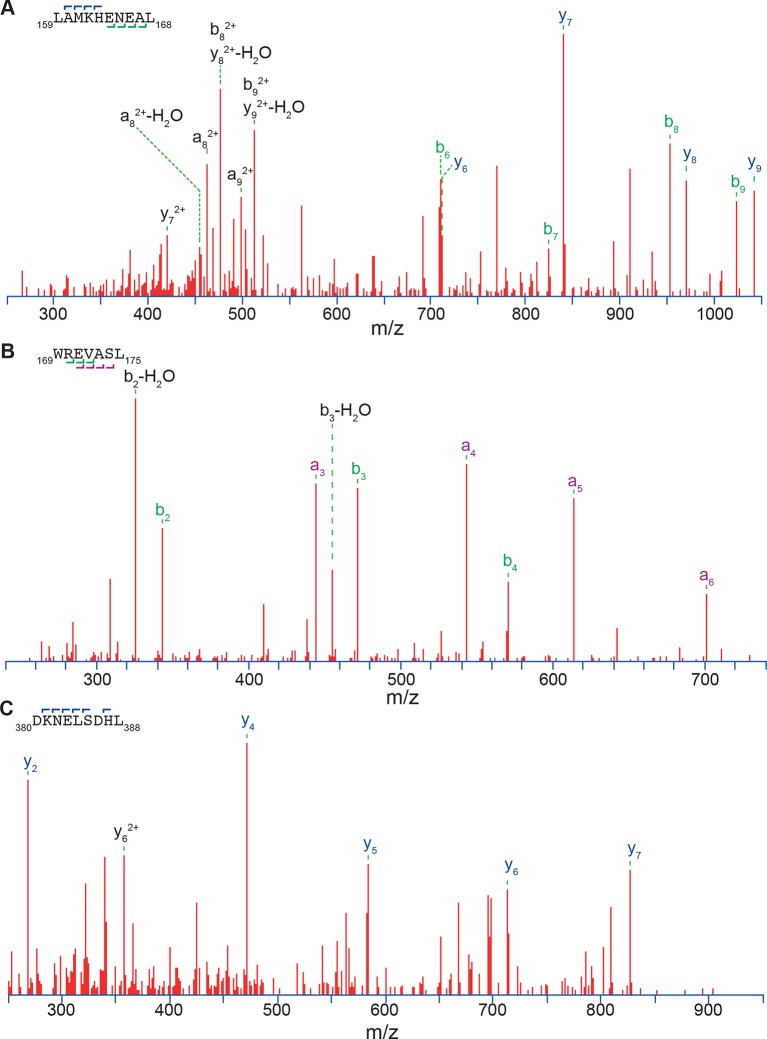
Three exemplary MS/MS spectra of peptic peptides used in the HX-MS analysis of Hsf1. **DOI:** http://dx.doi.org/10.7554/eLife.11576.008

**Figure 3.**
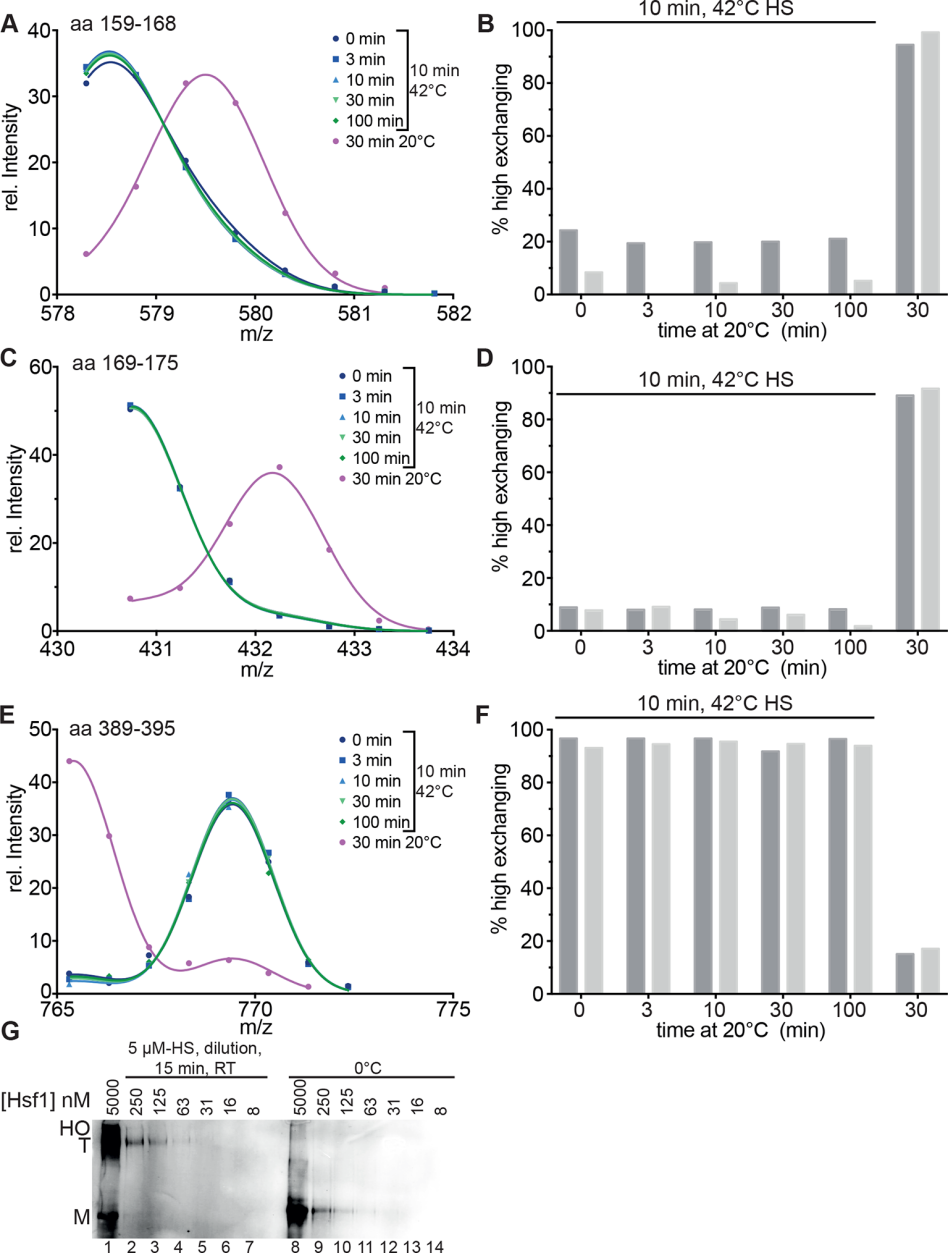
Heat-induced trimerization of Hsf1 is not reversible. (**A**–**F**) Prolonged incubation at 20°C does not revert the heat shock induced changes in Hsf1 conformation. Hsf1 (5 µM) was incubated for 10 min at 42°C and then shifted to 20°C. Aliquots were diluted at different time points (0, 3, 10, 30, 100 min) for 30 s into D_2_O buffer and subsequently analyzed by LC-MS. As control, Hsf1 was not heat-shocked and incubated for 30 min at 20°C before dilution into D_2_O. Shown are the intensity-m/z data for the indicated peptides from HR-A region (A, aa 159–168, 578.29^2+^; C, aa 169–175, 430.74^2+^) and region HR-C (E, aa 389–395, 765.31^1+^) with a global fit of an equation for two Gaussian peaks, as in Figure 2. The percentage of high exchanging species was calculated as described in Figure 2—figure supplement 2 (**B, D, F**). Data for two independent experiments are shown. (**G**) Dilution of heat-shocked Hsf1 does not lead to trimer dissociation. Hsf1 (5 µM) was heat-shocked at 42°C for 10 min, subsequently diluted as indicated, incubated at room temperature for 15 min, analyzed by blue-native gel electrophoresis, and detected by immune blotting with an human Hsf1 specific antiserum (lanes 1–7). Control samples were kept on ice before dilution and incubation at room temperature (lanes 8–14). M, monomer; T, trimer; HO, higher order oligomers (not detectable anymore upon dilution). **DOI:** http://dx.doi.org/10.7554/eLife.11576.009

We also determined the midpoint of transition for 30 min pre-incubation at different temperatures (Figure 2, Figure 2—figure supplement 3). Under these prolonged incubation conditions, the transition curves became more shallow, and the midpoint temperature for HR-C unfolding and HR-A protection (trimerization) was not identical anymore but decreased to 32.0 ± 0.4 and 34.7 ± 0.2°C, respectively. These data suggest that the temperature-induced conformational changes are not reversible under our conditions, otherwise the steepness of the curves, which is determined by the unfolding enthalpy, should remain the same as for the 10-min-pre-incubation. To investigate this in more detail we heat-shocked Hsf1 for 10 min at 42°C, then incubated the protein for different time intervals at 20°C, and analyzed the conformational state by HX-MS (Figure 3). As control, we incubated Hsf1 without prior heat shock for 30 min at 20°C before HX-MS analysis. We also tested whether dilution would lead to trimer dissociation in the time scale of our experiments (Figure 3G). Both assays clearly demonstrate that HR-C unfolding, HR-A protection and Hsf1 trimerization are not reversible under these conditions. Therefore, we cannot derive the unfolding enthalpy ∆H_U_ from our temperature response data but only use the fit to determine the temperature at which 50% of the transition occurred.

### Kinetics of temperature-dependent conformational changes

**Figure 4.**
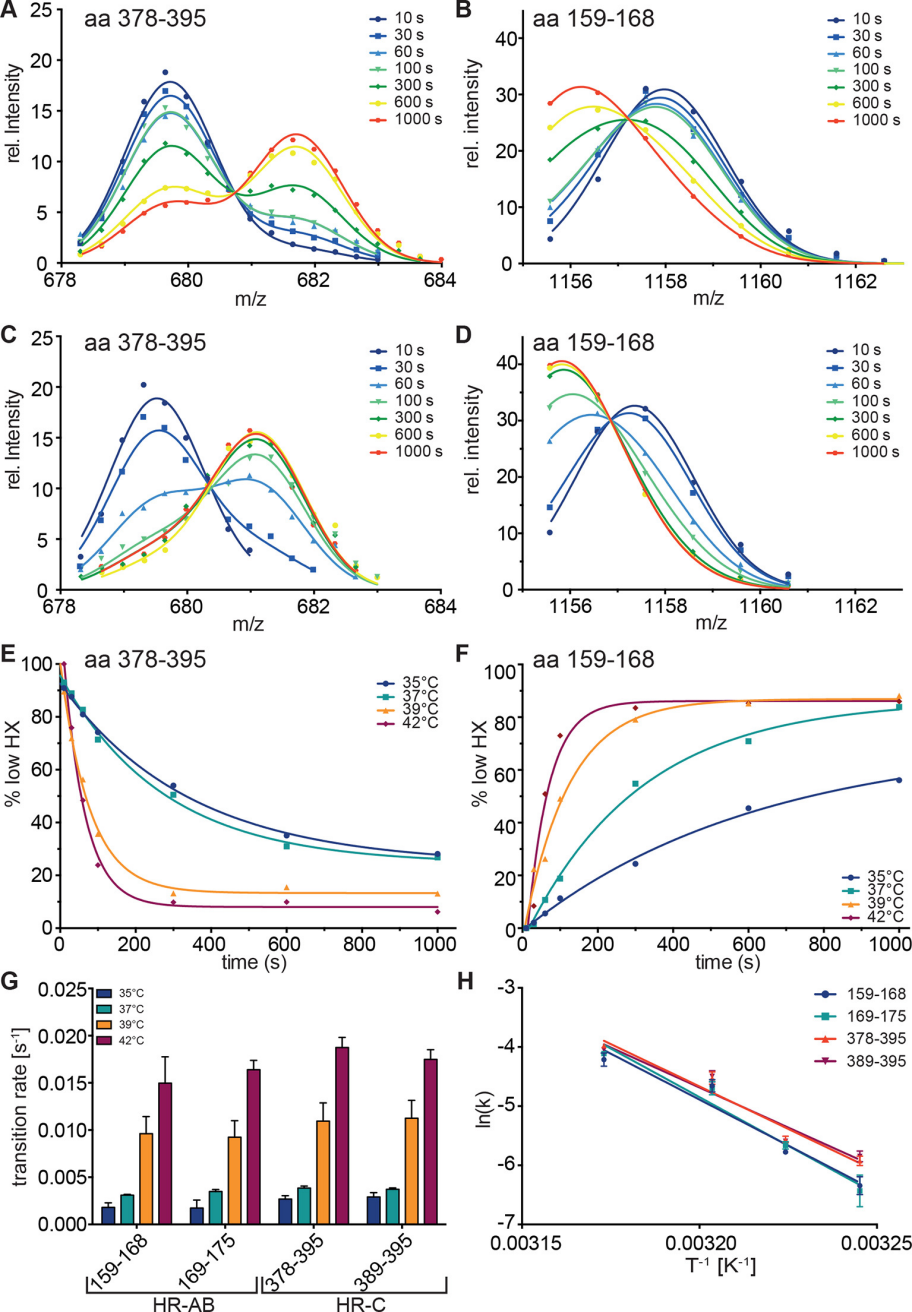
Kinetics of heat-induced conformational transitions in human Hsf1. (**A**–**D**) Intensity distributions of the isotope clusters for peptide 678.32^3+^ (aa 378–395) and 1155.58^1+^ (aa 159–168) of Hsf1 incubated at 35°C (**A** and **B**) or 42°C (**C** and **D**) for 10 to 1000 s. Curves are fits of the sum of two Gaussian peak functions to the data. (**E** and **F**) Change in the fraction of low exchanging species for peptides 378–395 (**E**) and 159–168 (**F**) for 35, 37, 39 and 42°C, as indicated. Curves are fits of a single exponential equation to the data. (**G**) Transition rates determined by fits as in panels **E** and **F** for all four peptides (159–168, 169–175, 378–395, 389–395) evaluated. (**H**) Arrhenius plot of the data shown in **G**. Linear regression analysis yielded an activation energy of 258 ± 25, 273 ± 26, 239 ± 19, and 225 ± 22 kJ·mol^-1^ for peptides 159–168, 169–175, 378–395, and 389–395, respectively. Error bars are SEM of three independent experiments. **DOI:** http://dx.doi.org/10.7554/eLife.11576.010

To resolve the kinetics of the conformational transitions, we pre-incubated monomeric Hsf1 for 10-1000 s at 35, 37, 39 or 42°C before diluting into D_2_O and incubation for 30 s at 20°C (Figure 4). For the peptic fragments of HR-C (amino acids 378–395 and 389–395) the low-exchanging population decrease with a rate of 0.0028 ± 0.0002, 0.0038 ± 0.0002, 0.011 ± 0.001, and 0.018 ± 0.001 s^-1^ at 35, 37, 39 and 42°C, respectively. For the peptic fragments of HR-A/B (amino acids 159–168 and 169–175) the low-exchanging population increased with slightly lower rates of 0.0018 ± 0.0004, 0.0033 ± 0.0002, 0.0094 ± 0.0009 and 0.016 ± 0.001 s^-1^ (Figure 4G). The Arrhenius plot of the data yielded the activation energy for the temperature transition of 249 ± 47 kJ·mol^-1^ (Figure 4H). Taken together, our data demonstrate that Hsf1 is a thermosensor that directly senses increasing temperatures with conformational changes in HR-A/B and HR-C.

### HR-C interacts with HR-A

**Figure 5.**
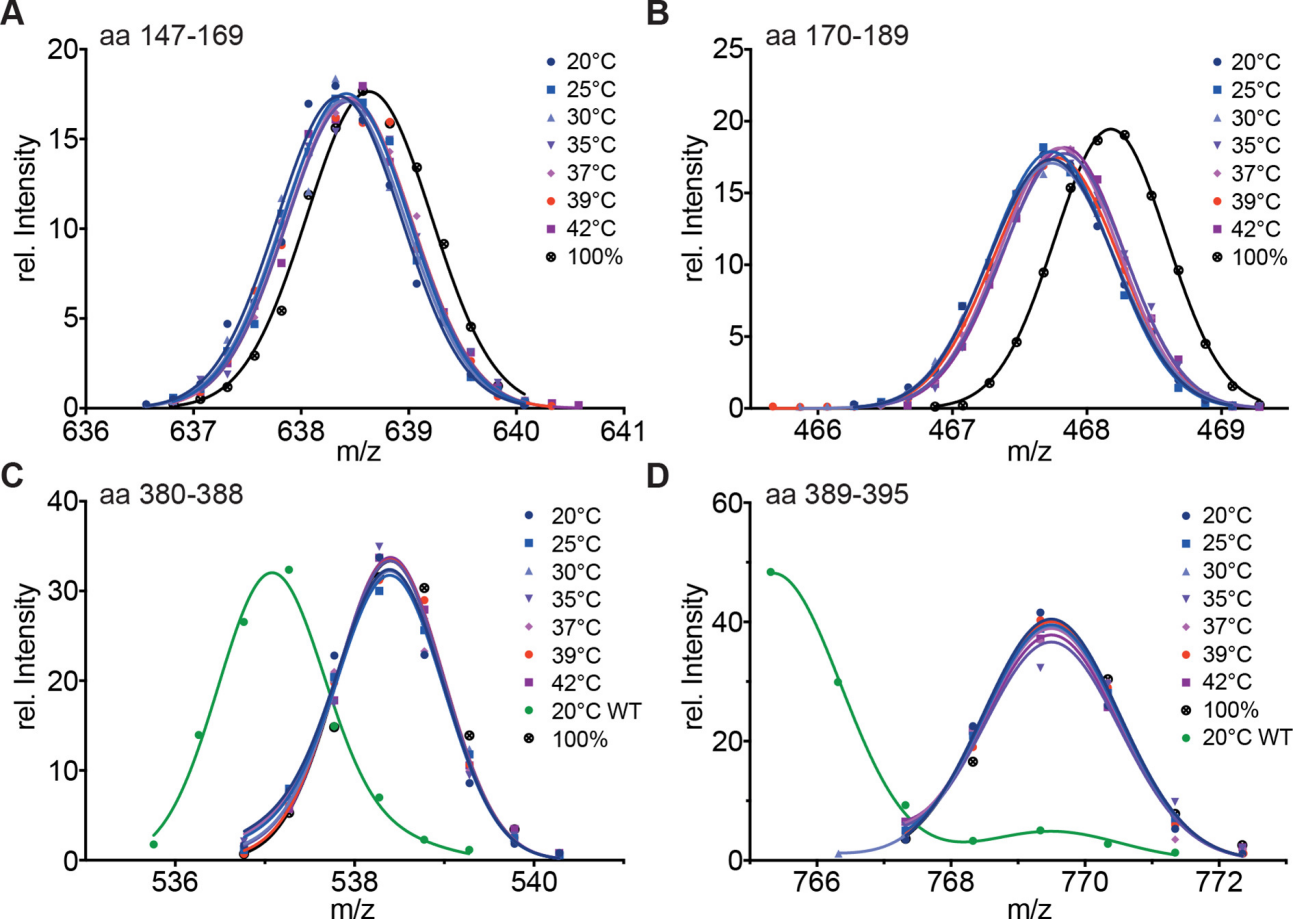
Hydrophobic residues in HR-A are essential for stability of HR-A and HR-C at all temperatures. (**A**–**D**) HX-MS analysis of Hsf1-I130S,V137S,L140S,V144S,M147S,M154S, L158S,M161S,L168S,V172S,L175S (Hsf1-HR-A-S11). Mutant protein was incubated at the different temperatures for 10 min and then analyzed by HX-MS. Shown are peptides from HR-A/B (**A**, aa 147–169, 634.80^4+^; **B**, aa 170–189, 465.26^5+^) and HR-C (**C**, aa 380–388, 535.76^2+^; **D**, aa 389–395, 765.31^1+^). For all peptides, the 100% deuterated control is shown and for the HR-C peptides a wild-type control, which was incubated for 30 min at 20°C to emphasize the decreased stability of the mutant protein. For the peptides from HR-A, no wild-type control peptides could be shown due to different sequence and cleavage by pepsin. Shown is one of three experiments with identical results. **DOI:** http://dx.doi.org/10.7554/eLife.11576.011

To uncouple the temperature-induced HR-C unfolding from trimerization, we replaced the hydrophobic heptad repeat residues in HR-A by serine, which should not engage in coiled-coil interactions. HR-A and HR-B are thought to be involved in trimerization, and HR-B had been previously been implicated in negative regulation of trimerization, since deletion of HR-B lead to continuous active trimeric HSF1 (Zuo et al., 1994). HX-MS experiments with the mutant protein revealed that HR-A/B and HR-C are constitutively unfolded at all temperatures tested (Figure 5). These results demonstrate that the ability of HR-A to form a coiled-coil is essential for stabilization of HR-A, HR-B and HR-C, suggesting that HR-C also interacts with HR-A.

### Mechanism of Hsf1 activation

**Figure 6.**
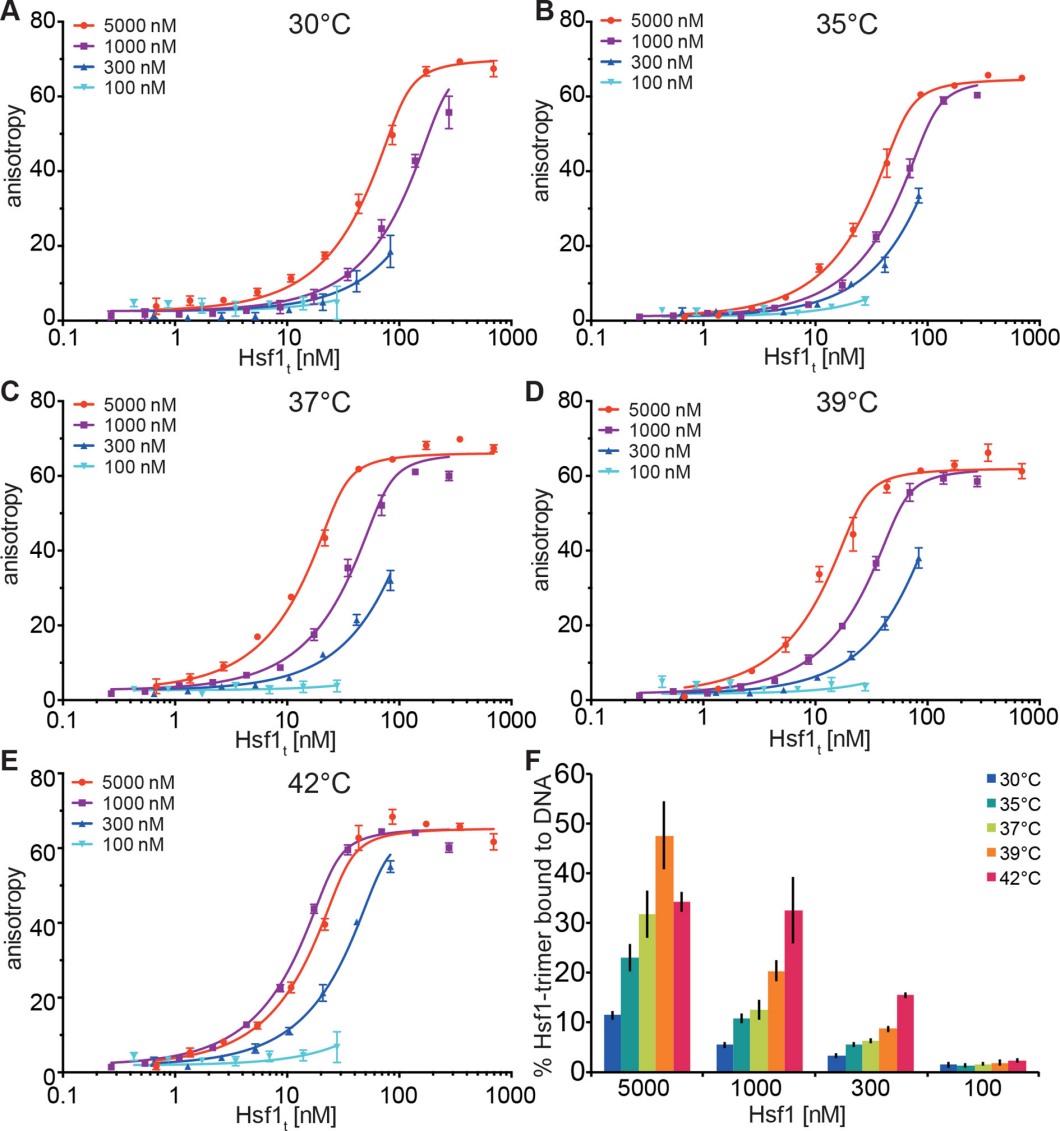
Temperature-induced acquisition of DNA-binding competence of Hsf1 is concentration dependent. (**A**–**E**) DNA-binding competence of monomeric Hsf1 after pre-incubation at the indicated temperature as measured by fluorescence anisotropy of 5’-Alexa 488-labeled HSE-DNA (^5’^ccccTTCccGAAtaTTCcccc^3’^). Monomeric Hsf1 was pre-incubated at 30-42°C at different concentrations (100–5000 nM) as indicated, then twofold dilution series were prepared and labeled DNA added. Plotted is fluorescence anisotropy (relative values) versus theoretical concentration of Hsf1 trimer. Curves represent a global fit of the quadratic solution of the binding equilibrium modified for fractional activity of Hsf1 to all data together resulting in a K_D_ of 1.10 ± 0.2 nM for fully active trimeric Hsf1 and a fraction of Hsf1 that formed DNA-binding competent trimers as shown in panel (**F**). Data of one representative experiment is shown. Error bars represent standard error of the mean of four technical replicates. (**F**) Fraction of Hsf1 that formed DNA-binding competent trimers at the given Hsf1 concentration and temperature as calculated from data in panels **A** to **E**. Mean and standard error of the mean of three independent sets of experiments are shown. **DOI:** http://dx.doi.org/10.7554/eLife.11576.012

In our in vitro experiments, the midpoint of trimerization of Hsf1 was around 36°C, which seemed rather low given a body core temperature of 37°C, and DNA binding activity of Hsf1 in different human cells is rather low below 40°C and strongly increased above 42°C (Abravaya et al., 1991; Mosser et al., 1990; Baler et al., 1993). In contrast, in testis the major increase in DNA binding activity of Hsf1 is already observed at 38°C (Sarge, 1995; Sarge et al., 1995). Therefore, the above described unfolding of HR-C cannot be the sole determinant for the setpoint of Hsf1 trimerization and DNA binding activity. Trimerization as multi-molecular reaction would be intrinsically concentration dependent. In contrast, inhibition of trimerization by coiled-coil interaction of HR-C with HR-A/B, as originally proposed by Wu and colleagues (Rabindran et al., 1993), would be an intramolecular reaction and consequently independent of concentration. Thus, the concentration of Hsf1 could be an important parameter for controlling the setpoint of the temperature response curve. Consistent with this hypothesis we noticed spontaneous trimerization of Hsf1 even at 4°C upon concentrating the protein. To explore this hypothesis more quantitatively, we devised a fluorescence anisotropy assay to determine the fraction of Hsf1 capable of binding DNA after treatment at different temperatures. We treated different concentrations of Hsf1 at 30, 35, 37, 39, and 42°C for 10 min, serially diluted the samples, incubated them with fluorescent labeled double-stranded DNA containing heat shock elements and measured fluorescence anisotropy (Figure 6). None of the formed Hsf1 trimers dissociated upon dilution and incubation at room temperature (Figure 3G). If Hsf1 trimerization were not concentration dependent in the concentration range tested, all data points would fall onto the same titration curve. This was obviously not the case. The quadratic solution of the binding equilibrium, modified for fractional active protein was globally fitted to the data, assuming identical K_D_ values for all formed Hsf1 trimers and similar minimum and maximum anisotropy values for no binding and complete binding, respectively. This fit yielded the fraction of DNA binding competent Hsf1 trimers. For 100 nM Hsf1, no activation was observed up to 42°C (Figure 6F). At 300 nM, less than 20% of the theoretical possible Hsf1 trimers had formed at 42°C within 10 min. In contrast, at 1 and 5 µM concentrations a substantial fraction of Hsf1 trimers had formed already at lower temperatures. At 42°C the apparent fraction of DNA-binding competent Hsf1 species may decrease due to the formation of high order oligomers as observed by blue native gel (Figure 1C).

**Figure 7.**
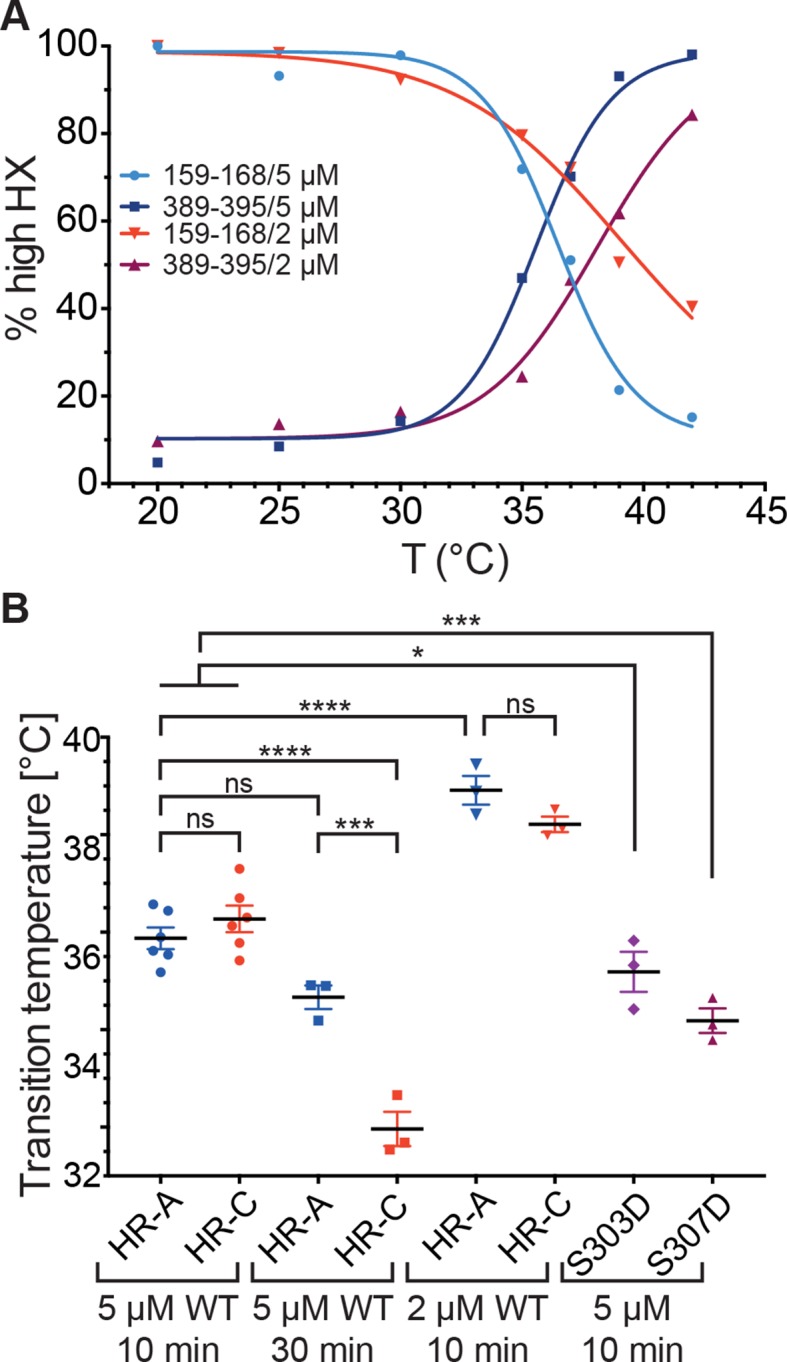
Temperature response curve of Hsf1 is concentration dependent. (**A**) Fraction of high exchanging species of peptides 159–168 and 389–395 for 5 and 2 µM Hsf1 pre-incubated at the respective temperatures and analyzed by HX-MS as in Figure 2. Data points and fits of the unfolding equilibrium equation of one representative of three independent experiments are shown. (**B**) Calculated midpoint temperature for wild-type Hsf1 (2 and 5 µM) and two phosphomimetic Hsf1 variants (5 µM). For 5 µM wild-type Hsf1 (10-min and 30-min-incubations) each data point represents the average of the T_m_ values for the two peptides observed in the respective region, which were not significantly different from each other (HR-A: aa 159–168 and 169–175; HR-C: 389–395 and 378–395 or 380–388). For 2 µM wild-type and for 5 µM mutant proteins no statistically significant differences were observed between the T_m_ values for the different peptides (159–168, 169–175, 378–395, 389–395) within an experiment, each data point represents the average of the T_m_ values of all evaluated peptides for an independent experiment. In addition to the data points of three to six independent experiments, the mean and standard error of mean is shown. *p<0.05; ***p<0.0005; ****p<0.0001; p-values were determined by Tukey’s multiple comparisons test. **DOI:** http://dx.doi.org/10.7554/eLife.11576.013

Since these data indicated that the temperature transition of Hsf1 is concentration dependent, we wondered whether only trimerization is affected or HR-C unfolding as well. We therefore repeated the HX-MS experiments at lower concentration (2 µM). Interestingly, not only trimerization but also unfolding of HR-C was concentration dependent and the differences between the transition temperatures of HR-A/B and HR-C were not statistically significant (Figure 7B). However, the difference in transition temperature between Hsf1 at 5 µM and at 2 µM was highly statistically significant (p<0.0001). We could not test lower concentrations of Hsf1 due to lacking sensitivity in the mass spectrometric detection of the important peptides.

Taken together, these data clearly demonstrate that HR-C unfolding, trimerization and the fraction of DNA-competent Hsf1 trimers are a function of temperature and concentration.

### Influence of phosphorylation on the thermosensor function

Human Hsf1 is heavily modified by posttranslational modifications (29 phosphorylation sites, 5 acetylation sites and 1 sumoylation site; www.phosphosite.org). Some of these modifications have been shown to influence Hsf1 activation (Holmberg et al., 2001; Guettouche et al., 2005; Xia et al., 1998; Xia and Voellmy, 1997; Chu et al., 1996; Wang et al., 2006; Soncin et al., 2003; Kim et al., 2005; Hietakangas et al., 2006; Westerheide et al., 2009; Raychaudhuri et al., 2014). Phosphorylation of Ser307 was proposed to negatively regulate the activation of Hsf1, because Hsf1-S307A was constitutively active in vivo (Chu et al., 1996; Xia et al., 1998). In contrast, phosphorylation of the close-by Ser303 had no influence on Hsf1 activation (Xia et al., 1998). Therefore, we constructed phosphomimetic variants of human Hsf1 (S307D and S303D as control) and determined their temperature response curves using HX-MS. The transition temperature for the phosphomimetic variants was slightly but statistically significantly lower than for Hsf1wt, indicating that phosphorylation at these sites does not prevent temperature-induced trimerization and might even aid it at physiological concentrations of Hsf1 (Figure 7B).

### Influence of Hsp90 on Hsf1 activation

**Figure 8.**
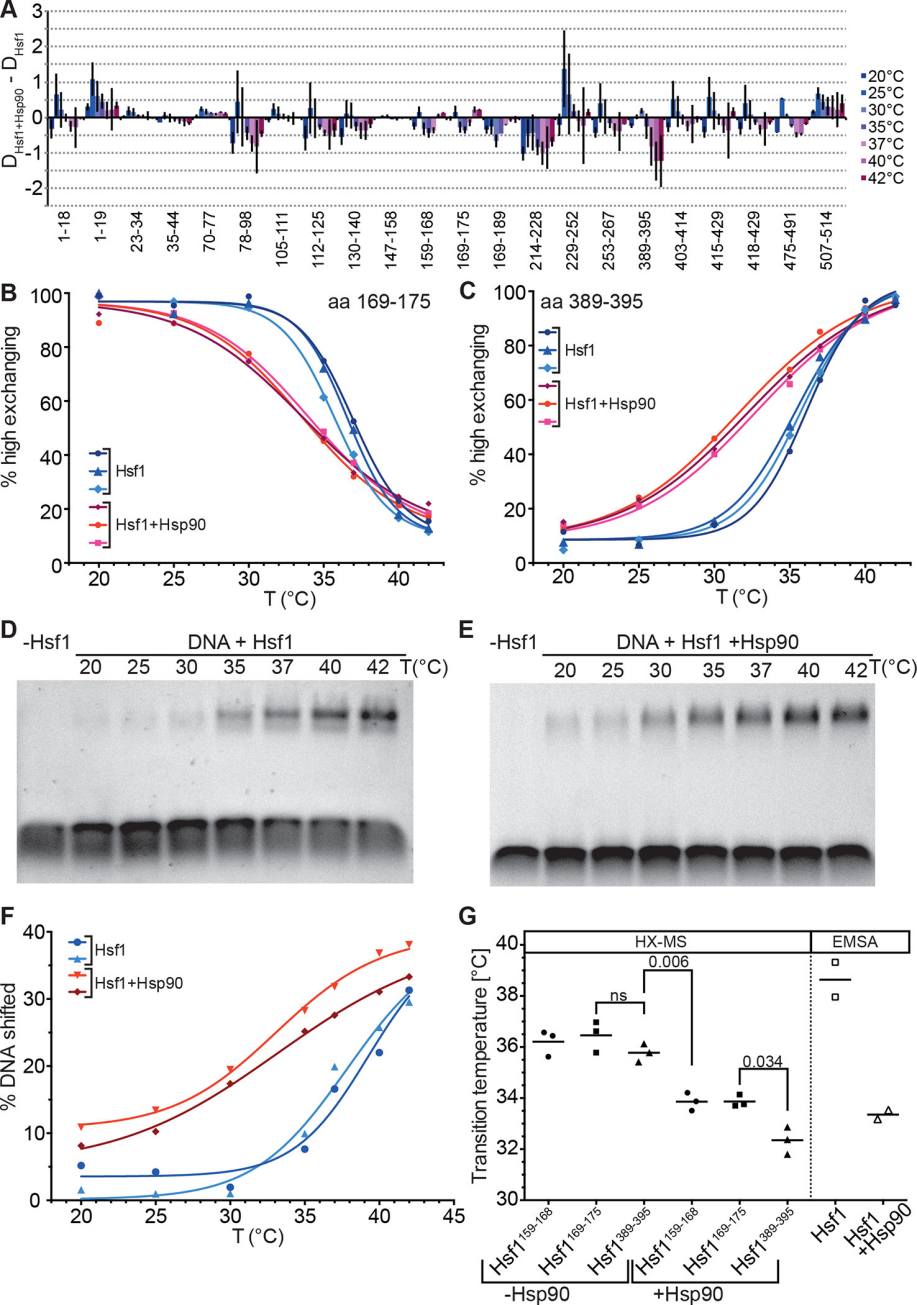
Hsp90 modulates midpoint and steepness of the temperature response curves of human Hsf1. (**A**) Difference plot of deuteron incorporation of human Hsf1 in the presence of Hsp90β minus deuteron incorporation into Hsf1 at the indicated temperatures. (**B** and **C**) Fraction of high-exchanging species of peptic peptides 169–175 (**B**) and 389–395 (**C**) of Hsf1 (5 µM) pre-incubated in the absence (blue) and presence of human Hsp90β (15 µM, red) at the indicated temperature before HX at 20°C for 30 s, quenching with low-pH buffer, peptic digestion, and MS analysis. Data points and fits of the unfolding equilibrium equation for three independent experiments are shown. (**D** and **E** Electrophoretic mobility shift assay (EMSA). HSE-DNA binding of monomeric Hsf1 pre-incubated at the indicated temperature in the absence (**D**) or presence of Hsp90 (**E**). (**F**) Quantification of data from panels **D** and **E**. Fraction of DNA bound by Hsf1 versus temperature is plotted. Data points and fits of the unfolding equilibrium equation to the data of two independent experiments are shown. (**G**) Transition midpoints calculated from the fits of panels **B, C**, and **F**. Numbers represent p values as determined by Tukey’s multiple comparisons test; ns, not significant. **DOI:** http://dx.doi.org/10.7554/eLife.11576.014

Several lines of evidence suggested that Hsp90 inhibits Hsf1 activation (Zou et al., 1998) and the current model assumes that Hsp90 binds Hsf1 in the monomeric state in unstressed cells (Anckar and Sistonen, 2011). We therefore studied the effect of human Hsp90β on the conformational dynamics of Hsf1, in particular the temperature response curve (Figure 8A–C). Surprisingly, in the presence of Hsp90 the midpoint of transition was lower than in its absence and the response curve was less steep, stretching the transition window from ~10° in the absence of Hsp90 to ~20°. Interestingly, the midpoint temperature of transition in the presence of Hsp90 was slightly lower for the peptide derived from HR-C than for the HR-A/B peptides (Figure 8G). A significant difference in midpoint temperature for the two regions was never observed in other experiments with 10-min-pre-incubation time but with a 30-min-pre-incubation at the different temperatures (compare Figure 8G with Figure 7B).

To verify that the effect of Hsp90 on trimerization is not an artifact of HX-MS methodology we performed electrophoretic mobility shift assays under comparable conditions using a fluorescently labeled DNA probe containing heat shock elements (Figure 8D–F). Within the experimental error, the results of the DNA binding assay were identical to those of the HX-MS experiments (Figure 8G). Taken together, our results demonstrate that in an in vitro assay with purified components, Hsp90 neither inhibits Hsf1 trimerization nor its DNA binding but, on the contrary, lowers transition temperature and widens the activation window.

## Discussion

In this study we demonstrate that human Hsf1 is a thermosensor. We are the first to show that the HR-C region of Hsf1 unfolds with temperature-dependent rates, resulting in a release of its repressive effect on Hsf1 trimerization and DNA binding. For relatively short heat shocks unfolding of HR-C and trimerization through intermolecular interactions of HR-A/B exhibit the same temperature response curves and follow identical kinetics, suggesting that these are coupled events. For longer heat shocks the cooperativity of the transition is reduced and HR-C unfolding and HR-A/B trimerization seem to uncouple. Most importantly, HR-C unfolding, temperature-induced trimerization and acquisition of DNA-binding competence depends on the concentration of Hsf1. Moreover, Hsp90 significantly modulated the temperature response of Hsf1, reducing midpoint and steepness of the response curve, thus widening the temperature window within which Hsf1 transits from monomer to trimer and low to maximal DNA binding competence. The response curves of Hsf1 in the presence of Hsp90 are similar to the response curves at prolonged incubation time at elevated temperatures. Thus, Hsp90 accelerates the response at intermediate heat shock conditions.

Our data with purified human Hsf1 are consistent with previous in vivo and in vitro work (Rabindran et al., 1993; Zhong et al., 1998; Baler et al., 1993; Zuo et al., 1994; 1995; Sarge et al., 1993; Zou et al., 1998), substantiating the hypothesis that Hsf1 trimerization and DNA binding is controlled by HR-C. Most previous work was performed in cellular systems or complex cell extracts and only few studies used purified components. Purified *Drosophila* Hsf1 was shown to exist in a trimer-monomer equilibrium that was influenced by temperature and oxidative stress (Zhong et al., 1998). However, human HSF1 does not seem to exist in such an equilibrium, as we did not observe dissociation of trimeric human Hsf1 upon dilution and heat-induced trimerization was irreversible in our hands (Figure 3). These data suggest that there are principle differences between *Drosophila* and human Hsf1.

For human Hsf1 we observed striking differences between the temperature response curves of 10 and 30-min-incubation at elevated temperatures. How can these differences be explained? Under our conditions temperature-induced Hsf1 transitions were irreversible. Therefore, at low temperatures even rare unfolding fluctuations of HR-C will eventually lead to trimerization, which will not be observed at short incubation times. Two effects could be responsible for the difference in T_m_ for HR-A/B and HR-C. At low temperature HR-C dissociation from HR-A/B and re-association might be fast as compared to trimerization. In the free state, HR-C could exist in an unfolding-refolding equilibrium, allowing exchange of protons for deuterons. If refolding is slow in comparison to the intrinsic chemical exchange rate, this would be visible as unfolded species, but HR-C would still refold and reassociate with HR-A/B, repressing trimerization. With increasing temperature unfolding rates would increase, shifting the equilibrium to the completely unfolded state, then allowing HR-A/B trimerization. Alternatively, additional temperature-induced conformational changes in HR-A/B are necessary to allow trimerization and these changes are slow as compared to HR-C unfolding at low temperatures.

Phosphorylation of Ser307 was suggested to repress human Hsf1 activation because the Ser307 to Ala replacement caused constitutively active Hsf1 in vivo (Xia et al., 1998). The temperature response curve of the phosphomimetic Hsf1-S307D variant measured by HX-MS showed a slightly reduced midpoint of transition as compared to wild-type Hsf1, suggesting that phosphorylation at this site does not inhibit heat-induced trimerization but might rather favor it and the repressive effect must be at a different level. Our observations are consistent with more recent data on non-phosphorylatable Hsf1 variants which were not constitutively active, casting a doubt on the repressive effect of phosphorylation at this site (Budzyński et al., 2015).

Most surprising was our finding that Hsp90 does not prevent trimerization and DNA binding of Hsf1 but in the contrary reduces midpoint and steepness of the temperature response curve. This observation seems to be at odds with the known repressive function of Hsp90 on the heat shock response (Zou et al., 1998; Whitesell et al., 2003; 2014; Sittler et al., 2001; Ali et al., 1998). This discrepancy may have different reasons. First, Hsp90 also inhibits the heat shock response in yeast, although yeast Hsf1 is constitutively trimeric and bound to DNA, suggesting that Hsp90 could exert its inhibiting function on human Hsf1 after trimerization and DNA binding as well, consistent with observations for human Hsf1 (Duina et al., 1998; Sorger et al., 1987; Guo et al., 2001). Second, in our experiments we only used one isoform of Hsp90, Hsp90β, and did not add any of the some 30 co-chaperones known to assist chaperoning by Hsp90. Further experiments with Hsp90α and different combinations of co-chaperones will be necessary to elucidate whether there exist isoform specificity in Hsf1 regulation or whether Hsp90-co-chaperone complexes have a different effect on Hsf1 trimerization than Hsp90β alone. Third, effects of Hsp90 down-regulation or inhibition could also be indirect, especially because interaction of Hsp90 with Hsf1 seems only to be observed after cross-linking (Neef et al., 2014; Zou et al., 1998), suggesting a very transient interaction. Hsp90 chaperones many kinases, and inhibition of Hsp90 leads to inactivation and degradation of these client proteins. Loss of such kinases could reduce inhibitory effects of phosphorylation or phosphorylation-dependent sumoylation of Hsf1 (Soncin et al., 2003; Wang et al., 2006; Hietakangas et al., 2003; 2006).

**Figure 9.**
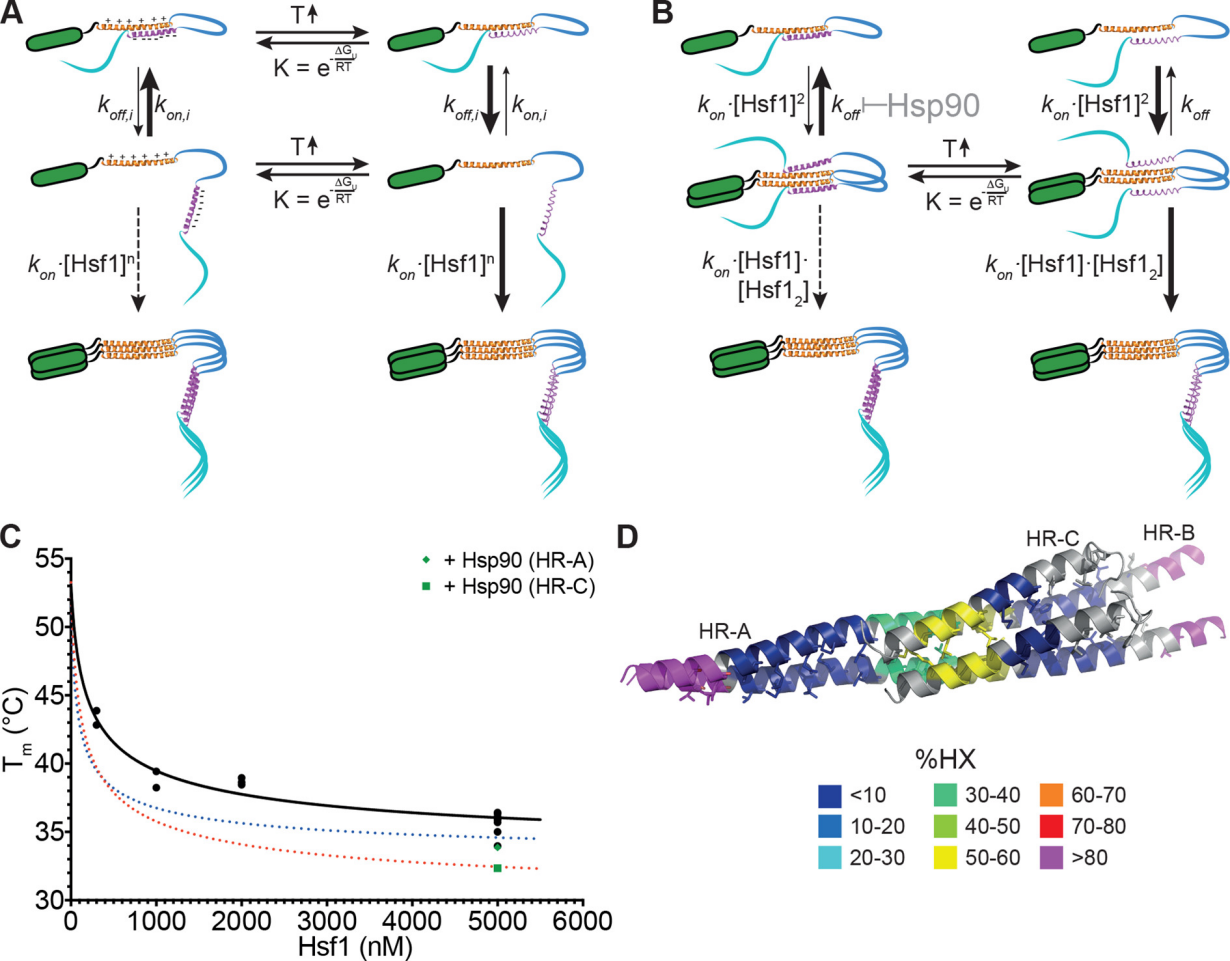
Kinetic models of the thermosensor function of Hsf1. (**A**) Monomer activation model, based on the originally proposed mechanism modified with our HX-MS data. In unstressed cells monomeric Hsf1 is in equilibrium between a closed, HR-C docked to HR-A/B, and open conformation, with HR-C dissociated from HR-A/B. Owing to high local concentration and electrostatic attraction the intramolecular association rate k_on,i_ of the HR-C–HR-A/B interaction are very high as compared to the dissociation rate k_off,i_. Since only uncomplexed HR-A/B can trimerize and Hsf1 trimerization therefore depends on the concentration of the open conformation, at low temperatures, trimerization only occurs at high Hsf1 concentrations. Temperature-induced unfolding of HR-C in the docked or undocked state reduces the intramolecular association rates and/or increases the dissociation rate of the intramolecular HR-C–HR-A/B complex, thereby increasing the concentration of Hsf1 in the open conformation and allowing trimerization at low Hsf1 concentrations. (**B**) Dimer activation model. At low temperatures, HR-C is constitutively docked onto HR-A/B and monomeric Hsf1 transiently dimerizes through the free part of HR-A/B. Such transient dimerization may partially destabilize the HR-C–HR-A/B interaction. At high Hsf1 concentrations a third Hsf1 monomer could interact with a transient Hsf1 dimer to form a thermodynamically stable Hsf1 trimer with completely released HR-C even at low temperatures. Increasing temperatures lead to unfolding of HR-C in the dimeric Hsf1 species leading to stabilization of the Hsf1 dimer and increased probability of trimerization. Hsp90 might modulate the temperature response by stabilizing the dimeric Hsf1 species. (**C**) Estimation of the concentration dependence of the transition temperature of Hsf1. Data points are all the T_m_ values determined for 10 min incubation at elevated temperatures for Hsf1 wild type in the absence (black) or presence (green) of Hsp90 by HX-MS and by anisotropy. Black curve is a fit of the quadratic solution of the law of mass action of the monomer-dimer equilibrium, assuming that the fraction of dimer determines the T_m_. This fit results in a T_m,M_ for the monomer of Hsf1 (extrapolation to 0 nM) of 53°C, the T_m,D_ for the dimer of 33°C, and a K_D_ of the monomer-dimer equilibrium of 330 nM. Due to the sensitivity of the fit to data points at low Hsf1 concentrations, these are only rough estimates. The blue and red dotted lines are simulations using a lower value for K_D_ (100 nM, blue) or K_D_ (200 nM) and T_m,D_ (29°C, red) to simulate the effect of Hsp90. (**D**) Tentative model of the dimeric Hsf1 based on the recent crystal structure of the trimerization domain of *C. thermophilum* Skn7, which formed tetramers in two different crystal forms (PDB ID 5D5Y and 5D5Z, [Neudegger et al., 2016]). HR-A, HR-B and HR-C were homology modeled on the tetrameric Skn7 using I-TASSER (Roy et al., 2010; Zhang, 2008; Yang and Zhang, 2015; Yang et al., 2015). HR-C was positioned to accommodate interactions with HR-A and HR-B. The homology model is colored according to HX-MS data (Figure 1E). Residues of the heptad repeat involved in the tetramer interface are shown as sticks. **DOI:** http://dx.doi.org/10.7554/eLife.11576.015

Originally, it was proposed that HR-C forms a coiled-coil with HR-A or HR-B to prevent trimerization in unstressed HSF1 (Rabindran et al., 1993; Zuo et al., 1994). Together with the observation that Hsf1 spontaneously trimerizes at high concentrations in the absence of a heat shock ([Zhong et al., 1998] and our own observations) and with our HX-MS data, showing temperature-induced unfolding of HR-C, the model shown in Figure 9A can be derived. Under non-stress conditions, HSF1 is in a conformational equilibrium between a closed conformation with HR-C interacting with HR-A/B and an open conformation, in which the two heptad repeat regions are dissociated. Since association of HR-C with HR-A/B is an intramolecular interaction, limiting the diffusional freedom of the interaction partners, association rates would be very high due to the apparent high local concentration. In addition, the net charge of HR-A/B and HR-C are +6 and -7, respectively, favoring association of the two regions by electrostatic attraction. Only at high concentrations of HSF1 association of HR-A/B regions of several HSF1 molecules, which are in the open conformation, would be able to compete with the intramolecular reaction, forming the thermodynamically more stable trimer. This would explain the spontaneous Hsf1 trimerization at high concentrations even at low temperatures (Zhong et al., 1998). Temperature-induced unfolding of HR-C in the closed conformation favors HR-C dissociation. Alternatively or in addition, HR-C unfolding in the open conformation prevents coiled-coil interaction with HR-A/B and thus reduces the back-reaction to the closed conformation. As a consequence, trimer association is favored even at lower HSF1 concentrations.

For two reasons we do not consider this model as very likely: First, single helices free in solutions are usually not stable but are in a rapid equilibrium with the unfolded state due to the low energy difference between helix-internal hydrogen bonds and hydrogen bonds with water. In such a state, we would not expect to see much protection in HX-MS experiments, which is in contrast to our observation (Figure 1E). The amount of heat necessary to unfold a single helix seems too small to account for the temperature control, the steepness, the kinetics and the substantial activation energy of 249 kJ·mol^-1^ for the unfolding/trimerization transition, observed in our experiments. Second, HR-C unfolding would be independent of concentration in this model, also inconsistent with our data (Figure 7).

Based on our data we propose a novel ‘dimer activation model’ (Figure 9B). In this model HR-C remains bound to HR-A/B in the unstressed Hsf1 monomer. However, unstressed Hsf1 could transiently dimerize due to the larger size of the HR-A/B region (75 amino acids) as compared to HR-C (42 amino acids). Such dimers would be destabilized by the interaction of HR-C with HR-A/B, resulting in high dissociation rates and a high fraction of Hsf1 monomers in unstressed cells. However, HR-A/B-HR-A/B interaction could also destabilize the interaction of HR-C with HR-A/B. At high Hsf1 concentrations, association of a third Hsf1 monomer to the transient Hsf1 dimer could occur, displacing the then more-weakly bound HR-C and leading to a stable Hsf1 trimer. Heat-induced unfolding of HR-C and possibly also its binding partner within HR-A/B (in our experiments obscured by subsequent trimerization) would lead to HR-C undocking. This would reduce Hsf1 dimer dissociation rates and favor Hsf1 trimerization even at low Hsf1 concentrations.

In this model, HR-C unfolding and trimerization are kinetically coupled processes, at least for short heat shocks (up to 15 min), explaining why they occur at identical rate constants (Figure 4). Furthermore, the average energy to unfold a protein which does not contain a co-factor is 1.4 kJ·mol^-1^ (Privalov and Gill, 1988; Alexander et al., 1992). Dividing the activation energy for HR-C unfolding determined by us, 249 kJ·mol^-1^, by 1.4 results in 175, suggesting that 175 residues are involved in this unfolding process. This is close to 168, the number of residues corresponding to a dimer of the coiled-coil between HR-C (42 residues) with a similar sized region in HR-A/B.

Our dimer activation model assumes that the proposed Hsf1 dimer has a lower T_m_ (T_m,D_) than the Hsf1 monomer and that the measured T_m_ depends on the fraction of Hsf1 dimer present in the assay. We therefore plotted all of our T_m_ values derived for wild-type Hsf1 and incubation times of 10 min versus Hsf1 concentration and fitted the quadratic solution of the law of mass action for the monomer-dimer equilibrium to the data (Figure 9C). This fit results in a K_D_ for the monomer-dimer equilibrium, the T_m,M_ for the monomer and the T_m,D_ for the dimer. However, the derived values are only very rough estimates due to the sensitivity of the curve to values of very low concentrations of Hsf1, which, for technical reasons, we could not determine so far. However, the steepness of the curve demonstrates that already small changes in concentration can dramatically change the transition temperature for Hsf1 activation.

Although there are no concentration determinations for Hsf1 in different tissues available to our knowledge, using the relative quantification data determined by mass spectrometry for 11 different cancer cell lines (Geiger et al., 2012), and assuming that these cells have a total protein concentration of about 150 mg/ml as determined for HEK293 cells (Gillen and Forbush, 1999), results in Hsf1 concentrations between 10 and 130 nM. The resulting T_m_ values would be between 47 and 53°C. However, Hsf1 is not equally distributed throughout the cytosol but shuttles in and out of the nucleus with various stress conditions preventing Hsf1 export out of the nucleus (Vujanac et al., 2005), resulting in a locally increased Hsf1 concentration between 4- and 17-fold (Fujioka et al., 2006) and a local concentration between 40 and 2210 nM. This is well within the range that would lead to a functional heat shock response according to our model.

Interestingly, the recent crystal structure of the trimerization domain of the Hsf1 homolog Skn7 of *Chaetomium thermophilum* contains trimers but also tetramers (PDB ID 5D5Z and 5D5Y, [Neudegger et al., 2016]). This tetramer might be a proxy for the dimer of HR-C-HR-A/B coiled-coils. To visualize how such an Hsf1 dimer might look like, we modeled the structure of HR-A, HR-B and HR-C using I-TASSER (Roy et al., 2010; Zhang, 2008; Yang and Zhang, 2015; Yang et al., 2015) and the tetramer structure of the trimerization domain of Skn7 as template (Figure 9D).

Hsp90 could modulate the monomer-trimer transition by stabilizing HR-A-HR-A interactions and/or destabilize HR-A/B-HR-C interactions, resulting in a reduced dimer dissociation rate and an increased rate of trimerization at lower temperatures. Stabilization of the HR-A-HR-A dimer and concomitant destabilization of HR-A/B-HR-C interaction would automatically destabilize HR-C ,since single helices are not stabile in solution and only stabilized by interaction with other structural elements, leading to a reduced unfolding transition temperature. To distinguish between stabilization of HR-A-HR-A interaction and destabilization of HR-A/B-HR-C interactions we simulated the effect of Hsp90 on the T_m_ by varying the apparent K_D_ for dimerization and/or the T_m,D_ of the dimer (see Figure 9C, dotted lines). With the current data available only changing the K_D_ does not reduce the T_m_ sufficiently to fit the measured values. However, small changes in K_D_ and T_m,D_ would give satisfying results. This would also explain the observation that Hsp90 reduced the T_m_ for HR-C unfolding significantly more than the T_m_ for trimerization. In this respect Hsp90, curiously, had a similar effect as the prolonged incubation at elevated temperatures (compare transition curves in Figure 2E and G and Figure 8B, C, and F). Thus, Hsp90 accelerates temperature-induced changes in conformation of Hsf1. It is not surprising that the chaperone Hsp90 destabilizes the HR-C conformation. Chaperones have been shown to locally unfold native proteins (Rodriguez et al., 2008; Sharma et al., 2010; Kirschke et al., 2014) and Hsp90 is believed to destabilize an α-helix in steroid hormone receptors to allow hormone binding.

How could Hsf1 be active at non-heat stress conditions, for example during development (Xiao et al., 1999), and how could it be activated by salicylate, low pH, Ca^2+^ ions, hypoxia, or proteotoxic stress other than heat shock as demonstrated previously (Mosser et al., 1990; Jurivich et al., 1992; Huang et al., 1995; Liu et al., 1996; Zhong et al., 1999; Ahn and Thiele, 2003)? According to our model Hsf1 exists in a monomer-dimer equilibrium, and trimerization of Hsf1 with subsequent DNA binding may occur continuously at low levels, promoted by Hsp90, as shown by us, and inhibited by TriC/CCT (Neef et al., 2014). This may ensure basal Hsf1 transcriptional activity under non-stress conditions. At the same time inhibition of Hsf1 activity by Hsp90, Hsp70-mediated attenuation and continuous Hsf1 monomerization would keep heat shock gene transcription at a low level. Any condition that would favor Hsf1 dimerization and thus trimerization or inhibit chaperone-mediated inhibition or attenuation would, as a consequence, increase heat shock gene transcription. All of the conditions mentioned above, including development, have been associated with an imbalance in proteostasis affecting Hsf1 through titrating away chaperones. However, Hsf1 dimerization could also be affected directly by changing its local concentration, as through transport of Hsf1 into the nucleus (Dai et al., 2003) or preventing its export (Vujanac et al., 2005), and by posttranslational modifications, including glutathionylation of the cysteine in HR-A in response to oxidative stress or alkylating agents (Liu et al., 1996), and phosphorylation of Thr142 in HR-A (Soncin et al., 2003), both of which would reduce the positive net charge of HR-A and thus the electrostatic repulsion.

Our model could explain why the temperature setpoint of activation was lower when human Hsf1 was expressed in *Drosophila* cells or in *Xenopus* oocytes (Baler et al., 1993; Clos et al., 1993). In transient or stable transfection experiments, usually a strong promoter is used to express the transfected gene. Thus, the concentration of Hsf1 might have been much higher in the transfected cell than is naturally the case in human cells. Similarly, in *Xenopus* oocyte-injection experiments the amount of injected mRNA determines the final concentration of HSF1 and might have been so high that the resulting Hsf1 concentration might have allowed activation already at 37°C. Finally, our kinetic Hsf1 activation model would allow each cell to adjust its setpoint of activation by changing the concentration of Hsf1 by producing more Hsf1 or by concentrating it in a smaller compartment, for example by transport from the cytoplasm into the nucleus. This would easily explain the different setpoints in testis (Sarge et al., 1995; Sarge, 1995), mouse T-lymphocytes (Gothard et al., 2003) and mouse motor neurons (Batulan et al., 2003). This might be particularly important for cancer cells for which it was shown that Hsf1 is a driver of malignancy (Dai et al., 2007).

## Materials and methods

### Protein purification

A culture of BL21 Rosetta, freshly transformed with a plasmid encoding the 6xHis-SUMO-Hsf1 wild-type or Hsf1-HR-A-S11 mutant sequence (Hsf1-I130S,V137S,L140S,V144S,M147S,M154S, L158S,M161S,L168S,V172S,L175S), was grown at 37°C to an OD_600_ of 0.6 and then shifted to 20°C. Expression was induced by addition of IPTG to a final concentration of 0.1 mM, the culture grown for 2 hr at 20°C and cells were subsequently harvested by centrifugation (4500 × g for 15 min). All following steps need to be carried out at 4°C. Cell pellets were resuspended in lysis buffer (25 mM Hepes pH 7.4, 150 mM NaCl and 10% glycerol, 3 mM β-mercaptoethanol) containing protease inhibitors (10 µg/ml aprotinin, 5 µg/ml leupeptin, 8 µg/ml pepstatin, one cOmplete Protease Inhibitor Cocktail tablet [Roche Diagnostics, Mannheim, Germany]). Cells were disrupted by subjecting the suspension two times to a chilled microfluidizer at a pressure of 1000 bar. The resulting lysate was immediately centrifuged (16000 × g for 45 min) to remove cell debris.

The supernatant fraction containing 6xHis-tagged HSF1 was incubated for 20 min at 4°C with 1 g of Protino Ni^2+^-IDA resin (Macherey-Nagel, Düren, Germany) in a rotation shaker. The resin was transferred to an empty gravity-flow column and the flow-through was collected. In a first step the resin was washed with 10 column volumes (CV) of wash buffer and 10 CV of high salt buffer (25 mM Hepes pH 7.4, 1 M NaCl, 10% glycerol, 3 mM β-mercaptoethanol). After a final washing step with another 10 CV of wash buffer the protein was eluted by addition of 1.5 CV elution buffer (25 mM Hepes pH 7.4, 1 M NaCl, 10% glycerol, 3 mM β-mercaptoethanol, 250 mM imidazole) to the column. The SUMO-Tag was cleaved off by incubation with Ulp1 SUMO-protease for 2 hr at 4°C.

The cleaved Hsf1 was further separated by size-exclusion on a S200 HiLoad 16/60 column (GE Healthcare Europe, Freiburg, Germany), equilibrated with Hsf1-buffer (25 mM Hepes pH 7.4, 150 mM NaCl, 10% glycerol, 2 mM DTT). The fractions containing monomeric Hsf1 were adjusted to a concentration of 10 µM, flash-frozen in liquid nitrogen and stored at -80°C.

### EMSA for trimerization of HSF1

300 nM Hsf1 premixed with 200 nM Cy3–labeled HSE-oligonucleotide were incubated for 10 min either on ice or at 42°C. As a positive control purified trimeric Hsf1 was kept on ice for 10 min. After incubation the samples were kept at room temperature for additional 30 min, mixed with glycerol and loaded onto a pre-chilled 1% agarose gel (TBE) at 4°C. The agarose gel was run for 30 min at 150 V in the cold room. Labeled HSE-DNA was detected on a FUJI LAS-4000 fluorescence imager (Fuji Photo Film, Düsseldorf, Germany).

For Hsp90 experiments, 5 µM Hsf1 was premixed with 2.5 µM Cy3–labeled HSE-oligonucleotide and 20 µM Hsp90β in buffer containing 10 mM ATP/20 mM MgCl_2_.

Samples were incubated for 10 min at different temperatures (20°C–42°C), diluted 1:6 in Hsf1-buffer and incubated for 30 min at room temperature. An amount of sample containing 850 nM Hsf1, 3.4 µM Hsp90β and 425 nM HSE-DNA was loaded onto a 1% agarose gel and processed as described above.

### HDX for temperature response curve

For the determination of the temperature response curve of Hsf1 activation, 5 µM Hsf1 or mutants (S303D or S307D) in Hsf1-buffer (25 mM Hepes pH 7.4, 150 mM NaCl, 10% glycerol, 2 mM DTT) were heat shocked for 10 or 30 min at different temperatures (20°C–42°C). The samples were then diluted 1:20 in D_2_O buffer and incubated for 30 s at 20°C. Deuterated samples were quenched 1:1 with ice-cold quench buffer (400 mM sodium phosphate pH 2.2), quickly injected into the injection valve and subjected to LC-MS using an Agilent UPLC and a MaXis mass spectrometer (Bruker, Bremen, Germany). For each experiment at least one unexchanged sample and one fully deuterated control was measured. To determine the Hsf1 activation temperature at lower concentrations, Hsf1 was diluted to a concentration of 2 µM before the experiment.

For HX-MS experiments in the presence of Hsp90β, 10 µM Hsf1 were mixed 1:1 with 40 µM human Hsp90β in reaction buffer (25 mM Hepes, 150 mM NaCl, 10% Glycerol, 20 mM MgCl_2_, 10 mM ATP and 2 mM DTT) and incubated for 10 min at 20°C. Equilibrated samples were then transferred to a thermomixer for a 10 min-incubation at seven different temperatures (20°C–42°C). Dilution in D_2_O buffer and subsequent steps were performed as described above.

The unexchanged protein sample was diluted 1:20 in H_2_O buffer and then mixed 1:1 with quench buffer. The fully deuterated sample (protein in Hsf1-buffer containing 6 M guanidine hydrochloride, lyophilised and redissolved in pure D_2_O at least three times) was treated equally to normal samples.

Data analysis was performed manually (Data Analysis 4.1, Bruker).

### HDX for kinetic studies

5 µM HSF1 in Hsf1-buffer were heat-shocked for different amounts of time (10 s, 30 s, 60 s, 100 s, 300 s, 600 s, 1000 s) at four different temperatures (35°C, 37°C, 39°C, 42°C). The samples were then diluted 1:20 in D_2_O buffer and incubated for 30 s at 20°C. Deuterated samples were quenched 1:1 with ice-cold quench buffer (400 mM sodium phosphate pH 2.2) and quickly injected into the injection valve and subjected to LC-MS. For each experiment at least one unexchanged sample and one fully deuterated control was measured.

Data analysis was performed manually (Data Analysis 4.1, Bruker).

### Data evaluation

Evaluation of bimodal isotope peak distribution: the intensity versus m/z plots of the isotope peaks were fitted with an equation for two Gaussian peaks (see Figures 2,4 or Figure 2—figure supplement 3):

I=A1σ·2π·e-12μ-μ1σ 2 + A2σ·2π·e-12μ-μ2σ2

with A_1/2_ being the area of the two peaks; μ, the m/z values; $\bar{\mu_{1/2}}$, the means of the Gaussian peaks, representing the centroid of each of the two subpopulations; and σ, the standard deviation of the Gaussian peaks, representing the width of the isotope peak distribution (see Figure 2—figure supplement 2B and D for individual Gaussian curves, the sum of which results in the fit curves of Figure 2). For each peptide showing a bimodal distribution all intensity values belonging to one temperature (Figures 2,3,5,7,8) or time (Figure 4) series was globally fitted assuming that σ, μ_1_ and μ_2_ are constant within this series. Independent experiments were treated independently. Then the parameters of the fit results, A_1/2_, $\bar{\mu_{1/2}}$ and σ, were used to calculate for each individual isotope peak which part of the intensity belongs to the low exchanging subpopulation and which part belongs to the high exchanging subpopulation (see Figure 2—figure supplement 2A and C panels: calculated intensity values for low [blue] and high [red] exchanging subpopulations for each isotope peak stacked on top of each other for comparison with original spectra). For all isotope peaks the intensities belonging to one subpopulation (low or high) was summed up to calculate the fraction of this subpopulation within the sample.

To calculate the temperature midpoint of the transition we used the thermal unfolding equation:

$$F=f_{0}+\left(f_{max}-f_{0}\right)∙\frac{e^{\frac{\left(T-T_{m}\right)∙\text{Δ}H}{R∙T∙T_{m}}}}{1+e^{\frac{\left(T-T_{m}\right)∙\text{Δ}H}{R∙T∙T_{m}}}}$$

with $f_{0}$ and $f_{max}$ being the fraction of high exchanging subpopulation at low and high temperatures, respectively; T, absolute temperature in K; T_m_, temperature at midpoint of activation; R, gas constant; ∆H, unfolding enthalpy.

### Blue native gel

Hsf1 (10 µM) in Hsf1-buffer were incubated for 30 min at 0°C (control) or 42°C (heat shock). Natively purified dimer and trimer of Hsf1 were added as additional controls. After incubation 7 µg of Hsf1 were loaded on a 7% native gel or a 4–16% native gradient gel and separated by blue native polyacrylamide gel electrophoresis as described in (Wittig et al., 2006) except that Coomassie Brillant Blue G250 was only present in the sample buffer (0.2%) not in the running buffer. For western blot analysis, an anti-Hsf1 antibody was used (Santa Cruz Biotech, HSF1 H-311).

### Fluorescence anisotropy

Aliquots of Hsf1 were thawed and immediately centrifuged (4°C, 15 min, 15000 rpm). In order to capture any occurring trimeric Hsf1, the supernatant was incubated for 20 min on ice with DNA containing three HSEs coupled to magnetic beads (5’-CCCCTTCCCGAATATTCCCCC-3’, 0.5 mg per aliquot, Dynabeads M-280 by Invitrogen). The supernatant concentration was determined by absorbance at 280 nm. Subsequently, four discrete concentrations of Hsf1 (5 µM, 1 µM, 300 nM, 100 nM) were prepared with Hsf1-buffer and heat-shocked for 10 min at five different temperatures (30°C, 35°C, 37°C, 39°C and 42°C) using a temperature-controlled water bath. Additionally, 300 nM Hsf1 was kept on ice for the same period of time as a control. Fluorescence anisotropy measurements were performed with a CLARIOstar microplate reader (BMG Labtech) and 384-well black flat-bottom microplates (Corning) in a final sample volume of 30 µL. Samples were serial diluted 1:2 until concentrations were below 1 nM. 10 nM of Alexa Fluor 488-labelled DNA containing three HSEs (5’-[A488]CCCCTTCCCGAATATTCCCCC-3’ (Sigma-Aldrich) was added by the injection system of the plate reader to start the measurement.

## References

[bib1] Abravaya K, Myers MP, Murphy SP, Morimoto RI (1992). The human heat shock protein hsp70 interacts with HSF, the transcription factor that regulates heat shock gene expression. Genes & Development.

[bib2] Abravaya K, Phillips B, Morimoto RI (1991). Attenuation of the heat shock response in HeLa cells is mediated by the release of bound heat shock transcription factor and is modulated by changes in growth and in heat shock temperatures. Genes & Development.

[bib3] Ahn SG, Thiele DJ (2003). Redox regulation of mammalian heat shock factor 1 is essential for hsp gene activation and protection from stress. Genes & Development.

[bib4] Alexander P, Fahnestock S, Lee T, Orban J, Bryan P (1992). Thermodynamic analysis of the folding of the streptococcal protein g IgG-binding domains B1 and B2: why small proteins tend to have high denaturation temperatures. Biochemistry.

[bib5] Ali A, Bharadwaj S, O’Carroll R, Ovsenek N (1998). HSP90 interacts with and regulates the activity of heat shock factor 1 in *xenopus* oocytes. Molecular and Cellular Biology.

[bib6] Anckar J, Sistonen L (2011). Regulation of HSF1 function in the heat stress response: implications in aging and disease. Annual Review of Biochemistry.

[bib7] Baler R, Dahl G, Voellmy R (1993). Activation of human heat shock genes is accompanied by oligomerization, modification, and rapid translocation of heat shock transcription factor HSF1. Molecular and Cellular Biology.

[bib8] Batulan Z, Shinder GA, Minotti S, He BP, Doroudchi MM, Nalbantoglu J, Strong MJ, Durham HD (2003). High threshold for induction of the stress response in motor neurons is associated with failure to activate HSF1. The Journal of Neuroscience.

[bib9] Brunet Simioni M, De Thonel A, Hammann A, Joly AL, Bossis G, Fourmaux E, Bouchot A, Landry J, Piechaczyk M, Garrido C (2009). Heat shock protein 27 is involved in SUMO-2/3 modification of heat shock factor 1 and thereby modulates the transcription factor activity. Oncogene.

[bib10] Budzyński MA, Puustinen MC, Joutsen J, Sistonen L (2015). Uncoupling stress-inducible phosphorylation of heat shock factor 1 from its activation. Molecular and Cellular Biology.

[bib11] Chu B, Soncin F, Price BD, Stevenson MA, Calderwood SK (1996). Sequential phosphorylation by mitogen-activated protein kinase and glycogen synthase kinase 3 represses transcriptional activation by heat shock factor-1. Journal of Biological Chemistry.

[bib12] Clos J, Rabindran S, Wisniewski J, Wu C (1993). Induction temperature of human heat shock factor is reprogrammed in a drosophila cell environment. Nature.

[bib13] Clos J, Westwood JT, Becker PB, Wilson S, Lambert K, Wu C (1990). Molecular cloning and expression of a hexameric drosophila heat shock factor subject to negative regulation. Cell.

[bib14] Dai C, Whitesell L, Rogers AB, Lindquist S (2007). Heat shock factor 1 is a powerful multifaceted modifier of carcinogenesis. Cell.

[bib15] Dai Q, Zhang C, Wu Y, McDonough H, Whaley RA, Godfrey V, Li HH, Madamanchi N, Xu W, Neckers L, Cyr D, Patterson C (2003). CHIP activates HSF1 and confers protection against apoptosis and cellular stress. The EMBO Journal.

[bib16] Duina AA, Kalton HM, Gaber RF (1998). Requirement for Hsp90 and a CyP-40-type cyclophilin in negative regulation of the heat shock response. Journal of Biological Chemistry.

[bib17] Fujioka A, Terai K, Itoh RE, Aoki K, Nakamura T, Kuroda S, Nishida E, Matsuda M (2006). Dynamics of the Ras/ERK MAPK cascade as monitored by fluorescent probes. Journal of Biological Chemistry.

[bib18] Geiger T, Wehner A, Schaab C, Cox J, Mann M (2012). Comparative proteomic analysis of eleven common cell lines reveals ubiquitous but varying expression of most proteins. Molecular & Cellular Proteomics.

[bib19] Gillen CM, Forbush B (1999). Functional interaction of the K-Cl cotransporter (KCC1) with the Na-K-Cl cotransporter in HEK-293 cells. The American Journal of Physiology.

[bib20] Gothard LQ, Ruffner ME, Woodward JG, Park-Sarge OK, Sarge KD (2003). Lowered temperature set point for activation of the cellular stress response in t-lymphocytes. Journal of Biological Chemistry.

[bib21] Graf C, Stankiewicz M, Kramer G, Mayer MP (2009). Spatially and kinetically resolved changes in the conformational dynamics of the Hsp90 chaperone machine. The EMBO Journal.

[bib22] Guettouche T, Boellmann F, Lane WS, Voellmy R (2005). Analysis of phosphorylation of human heat shock factor 1 in cells experiencing a stress. BMC Biochemistry.

[bib23] Guisbert E, Yura T, Rhodius VA, Gross CA (2008). Convergence of molecular, modeling, and systems approaches for an understanding of the escherichia coli heat shock response. Microbiology and Molecular Biology Reviews.

[bib24] Guo Y, Guettouche T, Fenna M, Boellmann F, Pratt WB, Toft DO, Smith DF, Voellmy R (2001). Evidence for a mechanism of repression of heat shock factor 1 transcriptional activity by a multichaperone complex. Journal of Biological Chemistry.

[bib25] Harrison C, Bohm A, Nelson H (1994). Crystal structure of the DNA binding domain of the heat shock transcription factor. Science.

[bib26] Hietakangas V, Ahlskog JK, Jakobsson AM, Hellesuo M, Sahlberg NM, Holmberg CI, Mikhailov A, Palvimo JJ, Pirkkala L, Sistonen L (2003). Phosphorylation of serine 303 is a prerequisite for the stress-inducible SUMO modification of heat shock factor 1. Molecular and Cellular Biology.

[bib27] Hietakangas V, Anckar J, Blomster HA, Fujimoto M, Palvimo JJ, Nakai A, Sistonen L (2006). PDSM, a motif for phosphorylation-dependent SUMO modification. Proceedings of the National Academy of Sciences of the United States of America.

[bib28] Holmberg CI, Hietakangas V, Mikhailov A, Rantanen JO, Kallio M, Meinander A, Hellman J, Morrice N, MacKintosh C, Morimoto RI, Eriksson JE, Sistonen L (2001). Phosphorylation of serine 230 promotes inducible transcriptional activity of heat shock factor 1. The EMBO Journal.

[bib29] Huang LE, Caruccio L, Liu AY, Chen KY (1995). Rapid activation of the heat shock transcription factor, HSF1, by hypo-osmotic stress in mammalian cells. Biochemical Journal.

[bib30] Jolly C, Morimoto RI (2000). Role of the heat shock response and molecular chaperones in oncogenesis and cell death. Journal of the National Cancer Institute.

[bib31] Jurivich D, Sistonen L, Kroes R, Morimoto R (1992). Effect of sodium salicylate on the human heat shock response. Science.

[bib32] Kim S-A, Yoon J-H, Lee S-H, Ahn S-G (2005). Polo-like kinase 1 phosphorylates heat shock transcription factor 1 and mediates its nuclear translocation during heat stress. Journal of Biological Chemistry.

[bib33] Kirschke E, Goswami D, Southworth D, Griffin PR, Agard DA (2014). Glucocorticoid receptor function regulated by coordinated action of the Hsp90 and Hsp70 chaperone cycles. Cell.

[bib34] Lee J-H, Gao J, Kosinski PA, Elliman SJ, Hughes TE, Gromada J, Kemp DM (2013). Heat shock protein 90 (hSP90) inhibitors activate the heat shock factor 1 (hSF1) stress response pathway and improve glucose regulation in diabetic mice. Biochemical and Biophysical Research Communications.

[bib35] Liu H, Lightfoot R, Stevens JL (1996). Activation of heat shock factor by alkylating agents is triggered by glutathione depletion and oxidation of protein thiols. The Journal of Biological Chemistry.

[bib36] Lu M, Kim H-E, Li C-R, Kim S, Kwak I-J, Lee Y-J, Kim S-S, Moon J-Y, Kim CH, Kim D-K, Kang HS, Park J-S (2008). Two distinct disulfide bonds formed in human heat shock transcription factor 1 act in opposition to regulate its DNA binding activity. Biochemistry.

[bib37] Morimoto RI (1998). Regulation of the heat shock transcriptional response: cross talk between a family of heat shock factors, molecular chaperones, and negative regulators. Genes & Development.

[bib38] Morimoto RI (2008). Proteotoxic stress and inducible chaperone networks in neurodegenerative disease and aging. Genes & Development.

[bib39] Mosser DD, Kotzbauer PT, Sarge KD, Morimoto RI (1990). In vitro activation of heat shock transcription factor DNA-binding by calcium and biochemical conditions that affect protein conformation. Proceedings of the National Academy of Sciences of the United States of America.

[bib40] Neef DW, Jaeger AM, Gomez-Pastor R, Willmund F, Frydman J, Thiele DJ (2014). A direct regulatory interaction between chaperonin TRiC and stress-responsive transcription factor HSF1. Cell Reports.

[bib41] Neudegger T, Verghese J, Hayer-Hartl M, Hartl FU, Bracher A (2016). Structure of human heat-shock transcription factor 1 in complex with DNA. Nature Structural & Molecular Biology.

[bib42] Pattaramanon N, Sangha N, Gafni A (2007). The carboxy-terminal domain of heat-shock factor 1 is largely unfolded but can be induced to collapse into a compact, partially structured state. Biochemistry.

[bib43] Powers MV, Clarke PA, Workman P (2008). Dual targeting of HSC70 and HSP72 inhibits HSP90 function and induces tumor-specific apoptosis. Cancer Cell.

[bib44] Powers MV, Workman P (2007). Inhibitors of the heat shock response: biology and pharmacology. FEBS Letters.

[bib45] Prahlad V, Cornelius T, Morimoto RI (2008). Regulation of the cellular heat shock response in caenorhabditis elegans by thermosensory neurons. Science.

[bib46] Prahlad V, Morimoto RI (2011). Neuronal circuitry regulates the response of caenorhabditis elegans to misfolded proteins. Proceedings of the National Academy of Sciences of the United States of America.

[bib47] Privalov PL, Gill SJ (1988). Stability of protein structure and hydrophobic interaction. Advances in Protein Chemistry.

[bib48] Rabindran S, Haroun R, Clos J, Wisniewski J, Wu C (1993). Regulation of heat shock factor trimer formation: role of a conserved leucine zipper. Science.

[bib49] Raychaudhuri S, Loew C, Körner R, Pinkert S, Theis M, Hayer-Hartl M, Buchholz F, Hartl FU (2014). Interplay of acetyltransferase EP300 and the proteasome system in regulating heat shock transcription factor 1. Cell.

[bib50] Rist W, Graf C, Bukau B, Mayer MP (2006). Amide hydrogen exchange reveals conformational changes in Hsp70 chaperones important for allosteric regulation. Journal of Biological Chemistry.

[bib51] Rodriguez F, Arsène-Ploetze F, Rist W, Rüdiger S, Schneider-Mergener J, Mayer MP, Bukau B (2008). Molecular basis for regulation of the heat shock transcription factor σ32 by the DnaK and DnaJ chaperones. Molecular Cell.

[bib52] Roy A, Kucukural A, Zhang Y (2010). I-TASSER: a unified platform for automated protein structure and function prediction. Nature Protocols.

[bib53] Sarge KD, Bray AE, Goodson ML (1995). Altered stress response in testis. Nature.

[bib54] Sarge KD, Murphy SP, Morimoto RI (1993). Activation of heat shock gene transcription by heat shock factor 1 involves oligomerization, acquisition of DNA-binding activity, and nuclear localization and can occur in the absence of stress. Molecular and Cellular Biology.

[bib55] Sarge KD (1995). Male germ cell-specific alteration in temperature set point of the cellular stress response. Journal of Biological Chemistry.

[bib56] Sharma SK, De Los Rios P, Christen P, Lustig A, Goloubinoff P (2010). The kinetic parameters and energy cost of the Hsp70 chaperone as a polypeptide unfoldase. Nature Chemical Biology.

[bib57] Shi Y, Mosser DD, Morimoto RI (1998). Molecularchaperones as HSF1-specific transcriptional repressors. Genes & Development.

[bib58] Sittler A, Lurz R, Lueder G, Priller J, Lehrach H, Hayer-Hartl MK, Hartl FU, Wanker EE (2001). Geldanamycin activates a heat shock response and inhibits huntingtin aggregation in a cell culture model of huntington's disease. Human Molecular Genetics.

[bib59] Soncin F, Zhang X, Chu B, Wang X, Asea A, Ann Stevenson M, Sacks DB, Calderwood SK (2003). Transcriptional activity and DNA binding of heat shock factor-1 involve phosphorylation on threonine 142 by CK2. Biochemical and Biophysical Research Communications.

[bib60] Sorger PK, Lewis MJ, Pelham HRB (1987). Heat shock factor is regulated differently in yeast and HeLa cells. Nature.

[bib61] Voellmy R (2004). On mechanisms that control heat shock transcription factor activity in metazoan cells. Cell Stress & Chaperones.

[bib62] Vuister GW, Kim S-J, Orosz A, Marquardt J, Wu C, Bax A (1994). Solution structure of the DNA-binding domain of drosophila heat shock transcription factor. Nature Structural Biology.

[bib63] Vujanac M, Fenaroli A, Zimarino V (2005). Constitutive nuclear import and stress-regulated nucleocytoplasmic shuttling of mammalian heat-shock factor 1. Traffic.

[bib64] Wang X, Khaleque MA, Zhao MJ, Zhong R, Gaestel M, Calderwood SK (2006). Phosphorylation of HSF1 by MAPK-activated protein kinase 2 on serine 121, inhibits transcriptional activity and promotes HSP90 binding. Journal of Biological Chemistry.

[bib65] Westerheide SD, Anckar J, Stevens SM, Sistonen L, Morimoto RI (2009). Stress-inducible regulation of heat shock factor 1 by the deacetylase SIRT1. Science.

[bib66] Whitesell L, Bagatell R, Falsey R (2003). The stress response: implications for the clinical development of Hsp90 inhibitors. Current Cancer Drug Targets.

[bib67] Whitesell L, Santagata S, Mendillo ML, Lin NU, Proia DA, Lindquist S (2014). HSP90 empowers evolution of resistance to hormonal therapy in human breast cancer models. Proceedings of the National Academy of Sciences of the United States of America.

[bib68] Wittig I, Braun H-P, Schägger H (2006). Blue native PAGE. Nature Protocols.

[bib69] Xia W, Guo Y, Vilaboa N, Zuo J, Voellmy R (1998). Transcriptional activation of heat shock factor HSF1 probed by phosphopeptide analysis of factor 32P-labeled in vivo. Journal of Biological Chemistry.

[bib70] Xia W, Voellmy R (1997). Hyperphosphorylation of heat shock transcription factor 1 is correlated with transcriptional competence and slow dissociation of active factor trimers. Journal of Biological Chemistry.

[bib71] Xiao X, Zuo X, Davis AA, McMillan DR, Curry BB, Richardson JA, Benjamin IJ (1999). HSF1 is required for extra-embryonic development, postnatal growth and protection during inflammatory responses in mice. The EMBO Journal.

[bib72] Yang J, Yan R, Roy A, Xu D, Poisson J, Zhang Y (2015). The i-TASSER suite: protein structure and function prediction. Nature Methods.

[bib73] Yang J, Zhang Y (2015). I-TASSER server: new development for protein structure and function predictions. Nucleic Acids Research.

[bib74] Zhang Y (2008). I-TASSER server for protein 3D structure prediction. BMC Bioinformatics.

[bib75] Zhong M, Kim S-J, Wu C (1999). Sensitivity of drosophila heat shock transcription factor to low pH. Journal of Biological Chemistry.

[bib76] Zhong M, Orosz A, Wu C (1998). Direct sensing of heat and oxidation by drosophila heat shock transcription factor. Molecular Cell.

[bib77] Zou J, Guo Y, Guettouche T, Smith DF, Voellmy R (1998). Repression of heat shock transcription factor HSF1 activation by HSP90 (hSP90 complex) that forms a stress-sensitive complex with HSF1. Cell.

[bib78] Zuo J, Baler R, Dahl G, Voellmy R (1994). Activation of the DNA-binding ability of human heat shock transcription factor 1 may involve the transition from an intramolecular to an intermolecular triple-stranded coiled-coil structure. Molecular and Cellular Biology.

[bib79] Zuo J, Rungger D, Voellmy R (1995). Multiple layers of regulation of human heat shock transcription factor 1. Molecular and Cellular Biology.

