# Supplementary material for: Molecular mechanism of thermosensory function of human heat shock transcription factor Hsf1
Source: eLife. 2016 Jan 19;5:e11576. doi: 10.7554/eLife.11576 (PMC4775227; doi:10.7554/eLife.11576)
Supplement: Related texts

# Article commentary: Decision letter

**DOI:** 10.7554/eLife.11576.016

**Affiliations:** University of Cambridge, United Kingdom

In the interests of transparency, eLife includes the editorial decision letter and accompanying author responses. A lightly edited version of the letter sent to the authors after peer review is shown, indicating the most substantive concerns; minor comments are not usually included.

Thank you for submitting your work entitled "Molecular mechanism of thermosensory function of human heat shock transcription factor Hsf1" for consideration by *eLife*. Your article has been reviewed by four peer reviewers, one of whom is a member of *eLife* Board of Reviewing Editors, and the process has been overseen by Michael Marletta as the Senior Editor.

The reviewers have discussed the reviews with one another and the Reviewing editor has drafted this decision to help you prepare a revised submission.

The merit of this paper is deemed to rest in the novel application of HDX to probe changes in the structure of mammalian HSF1 under different temperature and concentration conditions. Its key conclusion is that a temperature-induced change in the folded state of domain HR-C set in motion a chain of events that culminates in trimerisation and DNA binding. Thereby establishing a direct thermal sensing by HSF1 in its activation. Quantification of the temperature-dependence and concentration-dependence of changes in the folded state of HR-C and HR-AB provides fodder for plausible modeling of the effects of HSF1 concentration on its temperature-dependent activation profile (i.e. the driver of the heatshock response in mammals) and uncovered a surprising effect of Hsp90 on this dynamic process.

The reviewers have provided a wealth of suggestions as to how further improvements to the manuscript might be made and the authors are advised to look these over carefully (the unedited comments follow). But the consultation process has flagged the following as issues that must be addressed in the revised manuscript before acceptance. These issues were singled out for your attention because they are deemed to affect the strength of the papers conclusions:

1) Most of the experiments are based on H/D exchange. They reveal changes in local dynamics and accessibility depending on temperature variation. It is reasonable to assume that the defined changes identified by the authors are the critical changes that promote trimerization. However, as a proof of principle one would like to see an assay that reports directly on quaternary structure performed in parallel to some of the H/D experiments. Phrased differently, the kinetic data requires validation studies to correlate the changes observed in HX correlate with acquisition of HSF1 trimers and DNA binding activity.

2) The authors show in Figure 1C that monomeric Hsf1 forms trimers upon incubation at 42 degrees for 10 min, but did the authors also check what happens in the temperature range they used for HDX i.e. 30-45 degrees (the temperature region where a decrease in HX in HR-A/B is observed as shown in Figure 2D)?

3) The effect of Hsp90 observed vitro is a major surprise. The scope and significance of the paper would be expanded by learning if a reference chaperone (e.g. the Hsp70 system) has a similar effect.

4) While the authors present the baseline HX-MS data for control HSF1, the baseline data for fully activated HSF1would provider the reader with a sense of the dynamic range of the assay (i.e., the experimental results use 10 min exposures to the various temperatures but what are the HX-MS data for recombinant HSF1 exposed for prolonged times).

5) In fluorescence anisotropy experiments, the authors fitted all curves to the same equation assuming identical K_D_ values. However, for example, at 30 and 37/39/42 degrees, the shape of the red curve (5000 nM) appears different. At 30 degrees it is exponential while at 37/39/42 degrees, it is sigmoidal. Moreover, Hsf1 was incubated at different concentrations at different temperatures to enable trimer formation and later serially diluted. Did authors check whether the initial trimerization state of Hsf1 obtained during incubation at different temperatures is maintained upon serial dilutions?

6) The authors fitted the temperature response curves in the HDX experiments to the thermal equilibrium unfolding two state equation. An important parameter for the equilibrium unfolding fit is the reversibility of the reaction. Did authors check whether Hsf1 thermal unfolding is reversible?

*Reviewer #1:*

This paper presents a detailed characterization of the response of purified HSF1 to temperature changes as reflected in the HDX profile, oligomeric status, DNA binding affinity and the modulatory effect of Hsp90b on the latter parameter. The findings seem largely to corroborate the previously held views regarding the role of interactions between the C-terminal domain and the trimerisation domain of Hsf1 in repressing trimerisation and blocking inactivation. With temperature relieving the block.

The main surprise here is the effect of Hsp90, which is proven to lower the temperature threshold for trimerisation and attenuate the cooperativity observed in the transition from the repressed monomeric state to the active DNA-binding competent trimeric state. However this surprising result is not elaborated upon mechanistically nor is its significance to cell physiology explained.

The main merit of the paper thus lies in the significance of the application of HDX to this question and in the benefit to science from the carefully reasoned discussion of the quantitative parameters measured. This is a rather subjective matter best answered by experts in the field who can place the paper in context.

Here are some further suggestions from a non-expert:

The concordance between enhanced exchange of the HR-C peptide and the reduced exchange of the HR-AB peptide (Figure 2D and F) is consistent with the "thermosensor" being embodied in the transition between a state in which the two domains interact and the state in which HR-AB is trimeric and HR-C exposed to the solvent. It is thus unclear why the authors chose to describe the event as "unfolding" of the HR-C domain, for it seems to be equally relevant to describe it as unfolding of the HR-AB domain followed by its trimerisation. Can these two events be unlinked by mutagenesis: Are there mutations that preserve the AB-C interaction but block trimerisation; these might be predicted to promote temperature dependent enhanced exchange in both the AB and C regions?

The effect of Hsp90 on the HDX reactions (Figure 6A and B) has been carried out on the intact Hsf1. The apparent cooperativity between the effect of temperature on HDX by HR-AB and H-C limits the ability of these experiments to discriminate between the mechanisms proposed in Figure 7. But mutagenesis of Hsf1 has the potential to isolate the effects of Hsp90 to interactions with one domain or the other. Has this been tried?

Following melting of the AB-C (intramolecular) complex, oligomerisation competes with complex re-formation. Hence the concentration dependence of the relationship between Hsf1 concentration and the thermal sensing profile. Is it possible to model this relationship to predict the differences in Hsf1 needed to explain tissue-specific (testis versus other tissues) and heterologous cell type expression effects on temperature sensing? Are the predicted differences in Hsf1 concentration reasonable?

*Reviewer #2:*

The manuscript by Hentze et al. deals with a longstanding discussion on the mechanism of activation the heat shock transcription factor-1 (HSF1). The dogma in the field has been for long that the monomeric HSF-1 is kept inactive by chaperones (HSP90 and more recently also TRiC) until a proteotoxic stress occurs that titrates these chaperones to misfolded proteins, allowing HSF-1 to form trimers, the required step for it to activate the heat shock response (HSR). Even though generally cited, this model – or at least the generality of it – has been repeatedly questioned and the negative regulation by Hsp90 might likely be indirect. In this paper, direct evidence is provided that HSF1 itself undergoes heat-induced conformational changes that would suffice to explain heat-induced HSF1 activation, confirming earlier suggestions that HSF-1 in fact acts as the thermosensor.

Using recombinant HSF1 in vitro and hydrogen exchange MS (HX-MS) the authors show that two domains in HSF1 undergo temperature-induced conformational changes: 1) the HR-C in the transactivation domain shows increased HX (unfolding and thermosensor) followed by 2) the HR-A/B domain in the trimerization domain that shows decreased HX (consistent with trimer formation), indicating that the HR-C region is the thermosensor. Next, they show that HSF-1 concentration is an important parameter for the trimerization as well as for the DNA-binding competence of the trimer, which would be consistent with the possibility that HSF1 activation is different between species and cell types through adjustment of HSF-1 concentration.

Phosphorylation mimicking mutants of two serines (S303 and S307), which affect HSF-1 activity in cells, did not significantly affect these transition temperatures, meaning that these phosphorylations are not crucial for thermosensing.

Most intriguingly, also the addition of HSP90 to HSF-1 did not increase (but rather decreased) the transition temperature, implying that HSP90 is not a negative regulator of the thermosensing activity of HSF1. Hsp90 addition did broaden the transition temperature window, indicating that it does influence HSF1 activity.

Overall, the data are convincing and highly intriguing. The idea that the HR-C motif is crucial to HSF-1 trimerization and DNA binding (via intramolecular interactions with the HR-A/B domain) is not novel as also very correctly cited by the authors themselves. But, especially for the human HSF-1, the finding that it is the temperature sensing domain was never directly demonstrated before. The finding that Hsp90 is not inhibiting trimerization is the most surprising finding and challenges a long-lived dogma in the field. As stated again correctly by the authors themselves, it cannot be excluded that in vivo co-chaperones may still play a role, but this will have now to be shown in separate studies.

My question/comments that the authors may wish to consider are:

1) Whereas the data clearly demonstrate that Hsp90 (alone) does not negatively regulate HSF-1, I am somewhat puzzled the fin dings that HSP90 lowers the threshold temperature for HR-C unfolding. Also in both models presented in Figure 7, this does not become clear. Whilst in the dimer activation model, the effects of HSP90 on HR-A/B might be explained by assuming stabilization of the intermediate dimer, I do not understand how such would explain effects on HR-C unfolding. I further would like to add that I find Figure 7 and the related explanation (Discussion, fourth paragraph) not very clear in general.

2) Whilst the data strongly suggest HSF-1 as thermosensor and thus the main driver activation of the heat shock response at elevated temperature, HSF-1 can also be activated at physiological temperatures by various forms of proteotoxic stress. The existing model of negative regulation by Hsp90 or TRiC explained this by a shift of the chaperones from HSF-1 binding to binding to the stress-unfolded proteins. Can the authors speculate how they would interpret that data with their model and findings that Hsp90 does not negatively regulate HSF-1 activation?

*Reviewer #3:*

This manuscript addresses an important aspect of the regulation of the heat shock repsonse. In eukaryotic cells, the trimerization of the transcription factor Hsf1 is important for its activation. This process is affected by the molecular chaperones Hsp70 and Hsp90. The authors make excellent use of H/D exchange technology to obtain new insight on the underlying temperature-induced processes which finally lead to trimerization, including the influence of phosphorylation of Hsf1 and Hsp90 on the transition temperature of this process. Some technical issues need to be addressed.

Specific points:

1) Most of the experiments are based on H/D exchange. They reveal changes in local dynamics and accessibility depending on temperature variation. It is reasonable to assume that the defined changes identified by the authors are the critical changes that promote dimerization. However, as a proof of principle one would like to see an assay that reports directly on quaternary structure performed in parallel to some of the H/D experiments. This may also provide interesting information on the coordination of these events.

2) The authors show in Figure 1C that monomeric Hsf1 forms trimers upon incubation at 42 degrees for 10 min, but did the authors also check what happens in the temperature range they used for HDX i.e. 30-45 degrees (the temperature region where a decrease in HX in HR-A/B is observed as shown in Figure 2D)?

3) In Figure 2B no significant changes are observed in deuterium incorporation in the DBD at all the temperatures tested. I expected to see some changes in the DBD because trimerization of Hsf1 induces DNA binding and hence should cause certain conformational changes in the DBD to enable it to bind to DNA.

4) In fluorescence anisotropy experiments, the authors fitted all curves to the same equation assuming identical K_D_ values. However, for example, at 30 and 37/39/42 degrees, the shape of the red curve (5000 nM) appears different. At 30 degrees it is exponential while at 37/39/42 degrees, it is sigmoidal.

Moreover, Hsf1 was incubated at different concentrations at different temperatures to enable trimer formation and later serially diluted. Did authors check whether the initial trimerization state of Hsf1 obtained during incubation at different temperatures is maintained upon serial dilutions? Error bars should be shown in Figure 4.

5) In Figure 1C,a band is obtained at a size larger than the trimer. Does that mean that higher ordered oligomers are formed instead of trimers?

6) The authors fitted the temperature response curves in the HDX experiments to the thermal equilibrium unfolding two state equation. An important parameter for the equilibrium unfolding fit is the reversibility of the reaction. Did authors check whether Hsf1 thermal unfolding is reversible?

7) H/D exchange is temperature-dependent: the higher the temperature, the faster the exchange. In the experiments, Hsf1 was incubated at different temperatures and then diluted 1:20 at 20 degrees. The authors may want to add control experiments in which they show whether pre-incubation influences the exchange.

8) MS/MS spectra of the two peptides AA[159-168] and AA[389-395] discussed intensively could be added to a supplement. Furthermore, it may be useful to show difference plots for the effect of Hsp90 (similar to Figure 2B) at two different temperatures.

*Reviewer #4:*

The authors provide a potentially very interesting manuscript describing how Heat Shock Factor 1 might serve as a thermosensor for transducing a heat-stress signal in cells along with a role for the Hsp90 molecular chaperone in broadening the temperature response range of HSF1. They use an elegant biophysical approach (hydrogen-deuterium exchange coupled with mass spectrometry (HX-MS)) to delineate the polypeptide areas of HSF1 that are sensitive to temperature changes. Their work identifies a region that unfolds with temperature (HSF1 regulatory region) and an area that concomitantly tightens (trimerization region). The relative structural transitions in these regions in response to temperature are relatively sharp especially compared to the behavior in the presence of Hsp90, which broadens the structural transitions. In addition, the authors present data suggesting phosphorylation might also impact the responsiveness of HSF1 to temperature. In considering the work for publication the following points should be weighed:

1) Isolating monomeric, activatable HSF1 is challenging. The authors argue they have accomplished this feat. As this reagent is key to all of the presented findings it is imperative that is well characterized.

The basic model is that monomeric HSF1 will not bind DNA but heat stress will induce trimerization and a DNA binding activity. Yet, in Figure 1D there is clear DNA binding by what is supposed to be monomeric HSF1 since in lane 2 it has not been exposed to elevated temperature. Is this DNA binding resulting from spontaneous trimerization of their HSF1 or is monomeric HSF1 binding DNA? The authors should determine if HSF1 is trimerizing under their control conditions by incorporating a crosslinker and then probing for trimeric species of HSF1 by western blot analysis.

The presence of some trimeric HSF1 under control conditions is reasonable; however, the relative levels need to be determined and the presence of this species in their HX-MS studies needs to be considered.

2) While the authors present the baseline HX-MS data for control HSF1, the baseline data for fully activated HSF1 should also be given (i.e., the experimental results use 10 min exposures to the various temperatures but what are the HX-MS data for recombinant HSF1 exposed for prolonged times). Showing HX-MS data from equilibrated trimeric HSF1 will help in assessing the presenting kinetic activation data (Figure 3). In addition, the kinetic data requires validation studies to show that the changes they observe in HX correlate with acquisition of HSF1 trimers and DNA binding activity, which could be accomplished by including crosslinking and EMSA analysis of HSF1 exposed to similar conditions.

3) The authors present a potentially interesting concept that HSF1 concentration might influence its temperature activation properties (Figure 4). Given the novelty of this concept, in vivo data correlating with the in vitro work should be presented. For example, is HSF1 much more abundant in testis where its activation temperature is lower relative to other tissue types? Do all tissues with high levels of HSF1 display lower HSF1 activation responses? In addition, how do the shown concentrations of HSF1 compare to in vivo levels in any tissue type under normal or disease states? Changes in HSF1 concentrations in cancer cells would be especially interesting in light of the recent reports from the Lindquist laboratory showing constitutively active HSF1 in cancer cells.

4) The data generated with the phosphomimic mutants in Figure 5B are interesting. However, given the sensitivity of HX-MS it is quite possible that the amino acid substitutions are causing pleotropic effects. Hence, this work would require an assessment of actual phosphorylated HSF1 in order to be substantiated.

5) The work with Hsp90 is also intriguing. However, is this a general chaperone effect or is it specific to Hsp90? Would Hsp70 have a compare impact on the HX-MS data?

While the following is a bit outside the scope of this work, if we assume Hsp70 does impact HSF1 and given the authors focus on identifying factors that influence the HSF1 temperature set point, would the testis-specific Hsp70 have a distinct impact on HSF1 structural transitions relative to the more broadly expressed Hsp70s?

# Reply: Author response

**DOI:** 10.7554/eLife.11576.017

*[…] The reviewers have provided a wealth of suggestions as to how further improvements to the manuscript might be made and the authors are advised to look these over carefully (the unedited comments follow). But the consultation process has flagged the following as issues that must be addressed in the revised manuscript before acceptance. These issues were singled out for your attention because they are deemed to affect the strength of the papers conclusions: 1) Most of the experiments are based on H/D exchange. They reveal changes in local dynamics and accessibility depending on temperature variation. It is reasonable to assume that the defined changes identified by the authors are the critical changes that promote trimerization. However, as a proof of principle one would like to see an assay that reports directly on quaternary structure performed in parallel to some of the H/D experiments. Phrased differently, the kinetic data requires validation studies to correlate the changes observed in HX correlate with acquisition of HSF1 trimers and DNA binding activity.*

The reviewers are correct that our HX-MS data require validation by other methods that report on quaternary structure. For this reason we already had in the previous manuscript a blue native polyacrylamide gel showing that heating Hsf1 for 10 min at 42°C leads to a slower migrating band of trimeric Hsf1 (Figure 1C). We also showed electrophoretic mobility shift assays (EMSA) reporting on DNA binding, which increased dramatically after heating to 42°C (Figure 1D). It has been shown before that monomeric Hsf1 has a very low affinity to its cognate DNA motif and needs to trimerize to detect DNA binding by EMSA (Baler et al., 1993). We also showed in the previous manuscript a temperature response curve for Hsf1 measured by EMSA (Figure 6C). We agree that this control is rather late in the manuscript but we wanted to show these data next to the temperature response curve for Hsf1 in the presence of Hsp90β to allow immediate comparison.

In the revised manuscript we have now included a new temperature response curve for Hsf1 followed by blue native polyacrylamide gel electrophoresis, reporting directly on the change in quaternary structure in response to pre-incubation at different temperatures (new Figure 2B and Figure 2—figure supplement 1).

*2) The authors show in Figure 1C that monomeric Hsf1 forms trimers upon incubation at 42 degrees for 10 min, but did the authors also check what happens in the temperature range they used for HDX i.e. 30-45 degrees (the temperature region where a decrease in HX in HR-A/B is observed as shown in Figure 2D)?*

These data are now provided in new Figure 2B and Figure 2—figure supplement 1. We added to the manuscript:

“As control we analyzed the pre-treated Hsf1 by blue native polyacrylamide gel electrophoresis (Wittig et al., 2006) and observed temperature dependent increase in trimeric Hsf1 species (Figure 2B and Figure 2—figure supplement 1).”

*3) The effect of Hsp90 observed vitro is a major surprise. The scope and significance of the paper would be expanded by learning if a reference chaperone (e.g. the Hsp70 system) has a similar effect.*

We would like to thank the editor for waving this point. As explained in a letter to the editor, extending our study performed with Hsp90 to another chaperone (e.g. Hsp70), is much more complex than the reviewers seem to realize. First, addition of another protein in HX-MS experiments requires a new establishment of the analysis. Since the chaperone is added in several fold excess and, depending on the digestion efficiency of the chaperone itself, peptides will overlap with Hsf1 peptides, the liquid chromatography gradient has to be adjusted to be able to separate the relevant peptides. Second, to give meaningful results Hsp70 should not be used alone but should be used in combination with one of the some 16 J-domain co-chaperones predicted to be present in the cytosol of human cells and maybe one of the seven nucleotide exchange factors. At present we have no idea which of the J-domain proteins and which of the nucleotide exchange factors is relevant for Hsf1 regulation. Also, should the heat-inducible Hsp70 or the constitutive Hsc70 be used in such a study? We think this is a completely new study that will be interesting but in our opinion goes far beyond the current manuscript. Besides, Hsp70 was shown not to affect activation of Hsf1 but attenuation (Rabindran et al. 1994 Mol Cell. Biol). I am not sure how such a study would impact our results with Hsp90. If Hsp70 has a similar effect or not, will not substantiate our findings with Hsp90.

*4) While the authors present the baseline HX-MS data for control HSF1, the baseline data for fully activated HSF1would provider the reader with a sense of the dynamic range of the assay (i.e., the experimental results use 10 min exposures to the various temperatures but what are the HX-MS data for recombinant HSF1 exposed for prolonged times).*

Already in the previously submitted manuscript we provided data on Hsf1 pre-incubation at four different temperatures for extended times (previous Figure 3, now Figure 4). We have now followed the reviewers’ suggestion and determined temperature response curves with 30 min pre-incubation at the different temperature. As already suggested by our previous kinetic data, the transition midpoint temperature for trimerization decreased albeit only slightly from 36.15 to 34.7°C. Interestingly, the transition curves for 30 min incubation were less steep and the unfolding transition for HR-C was significantly lower at 32°C. These data are now included in Figure 2E and G and are discussed.

*5) In fluorescence anisotropy experiments, the authors fitted all curves to the same equation assuming identical K_D_ values. However, for example, at 30 and 37/39/42 degrees, the shape of the red curve (5000 nM) appears different. At 30 degrees it is exponential while at 37/39/42 degrees, it is sigmoidal.*

All data points of all concentrations and all temperatures were globally fitted together with the quadratic solution of the law of mass action, keeping the K_D_, lower and upper anisotropy limits constant for all data, resulting in sigmoidal curves on a logarithmic scale. Therefore, the 30°C curve only appears exponential but is in reality part of a sigmoidal curve. The global fit was necessary since, as the reviewers observe correctly, at some temperatures not enough trimeric Hsf1 is generated to saturate DNA-binding and reach the upper limit of the anisotropy values and therefore the upper limit is not defined for these temperatures. Fitting the data individually would not yield a K_D_ or upper limit. But assuming that complete binding of the DNA always results in the same anisotropy value, the saturation data of the higher temperature series can be used to define the upper limit of the sigmoidal curve for the lower temperature series.

*Moreover, Hsf1 was incubated at different concentrations at different temperatures to enable trimer formation and later serially diluted. Did authors check whether the initial trimerization state of Hsf1 obtained during incubation at different temperatures is maintained upon serial dilutions?*

The reviewers raise an important issue for these experiments. Already in the previous version of the manuscript we had mentioned in the Discussion section that trimerization of human Hsf1 was irreversible under the conditions we have tested. In the revised version of the manuscript we provide conclusive evidence that under the conditions used in our fluorescence anisotropy experiments Hsf1 trimerization is irreversibel. We converted Hsf1 into the trimeric form by heating 5 µM monomeric Hsf1 for 10 min to 42°C. We subsequently diluted the sample and incubated them at room temperature for 15 min. On an immunoblot of a blue native polyacrylamide gel we do only detect trimeric Hsf1 in the diluted samples but no Hsf1 monomers (Figure 3G). We added to the manuscript:

“None of the formed Hsf1 trimer dissociated upon dilution and incubation at room temperature (new Figure 3).”

*6) The authors fitted the temperature response curves in the HDX experiments to the thermal equilibrium unfolding two state equation. An important parameter for the equilibrium unfolding fit is the reversibility of the reaction. Did authors check whether Hsf1 thermal unfolding is reversible?*

The reviewers touch a sensitive point. As mentioned above, Hsf1 trimerization was not reversible under our conditions. We now show by HX-MS that HR-C does not refold during prolonged incubation at room temperature after a 10-min heat shock at 42°C (new Figure 3). We also performed serial dilutions of heat-shocked Hsf1, incubated these samples at room temperature, and analyzed Hsf1 trimer by blue native gel electrophoresis (new Figure 3G). Although we fitted the thermal equilibrium unfolding equation to our temperature response data, we used the fits only to determine the midpoint temperature for the transition and not to determine ∆H, which is only possible, if the reaction is fully reversible within the time scale of the experiment. This is now stated more clearly in the revised version of the manuscript:

“To investigate this [reversibility of trimerization] in more detail we heat shocked Hsf1 for 10 min at 42°C, then incubated the protein for different time intervals at 20°C, and analyzed the conformational state by HX-MS (Figure 3). […] Therefore, we cannot derive the unfolding enthalpy ∆H_U_ from our temperature response data but only use the fit to determine the temperature at which 50% of the transition occurred.

Of note, the analyzed peptides occurred in different charge states, the relative intensities of which varied between experiments. We always analyzed and show the charge state with the highest intensity, which gives the most accurate result. For this reason the m/z scales for identical peptides may differ in different figures. We only point this out to avoid confusion.

*Reviewer #1: This paper presents a detailed characterization of the response of purified HSF1 to temperature changes as reflected in the HDX profile, oligomeric status, DNA binding affinity and the modulatory effect of Hsp90b on the latter parameter. The findings seem largely to corroborate the previously held views regarding the role of interactions between the C-terminal domain and the trimerisation domain of Hsf1 in repressing trimerisation and blocking inactivation. With temperature relieving the block.*

We would like to point out that the molecular mechanism of Hsf1 trimerization that we show in our study and illustrate in previous Figure 6B (now Figure 8B) is very different from previous models. Since unfolding of HR-C depends on the concentration of HSF1, as we demonstrate in our study, the simple model proposed by Wu and colleagues cannot be operative. In such a model the relief of the repressive function of HR-C on Hsf1 trimerization would not depend on the concentration of Hsf1. There might have been before circumstantial evidence for a concentration dependence of Hsf1 activation but the mechanistic implications of such incidental observations have not been realized.

*The main surprise here is the effect of Hsp90, which is proven to lower the temperature threshold for trimerisation and attenuate the cooperativity observed in the transition from the repressed monomeric state to the active DNA-binding competent trimeric state. However this surprising result is not elaborated upon mechanistically nor is its significance to cell physiology explained.*

As the reviewer might realize, providing mechanistic insights into this phenomenon and giving cell physiological explanations is far from trivial and requires a new study, which we will undertake as soon as possible.

*The main merit of the paper thus lies in the significance of the application of HDX to this question and in the benefit to science from the carefully reasoned discussion of the quantitative parameters measured. This is a rather subjective matter best answered by experts in the field who can place the paper in context. Here are some further suggestions from a non-expert:*

*The concordance between enhanced exchange of the HR-C peptide and the reduced exchange of the HR-AB peptide (Figure 2D and F) is consistent with the "thermosensor" being embodied in the transition between a state in which the two domains interact and the state in which HR-AB is trimeric and HR-C exposed to the solvent. It is thus unclear why the authors chose to describe the event as "unfolding" of the HR-C domain, for it seems to be equally relevant to describe it as unfolding of the HR-AB domain followed by its trimerisation.*

The reviewer is possibly correct that not only HR-C is unfolding but also HR-A or HR-B or both followed by trimerization of HR-AB. We do not directly observe unfolding of either HR-A or HR-B. Even at the 30 min incubation (included in the revised manuscript), which showed a lower transition temperature for HR-C than for trimerization, we did not observe any indication of further HR-A or HR-B unfolding. Therefore, we preferred to state in our manuscript what is directly observed. However, we added to the Discussion section that the interaction partner of HR-C within HR-A/B may also unfolded during the transition to the trimeric state:

“Heat-induced unfolding of HR-C and possibly also its binding partner within HR-A/B (in our experiments obscured by subsequent trimerization) would lead to HR-C undocking.”

Can these two events be unlinked by mutagenesis: Are there mutations that preserve the AB-C interaction but block trimerisation; these might be predicted to promote temperature dependent enhanced exchange in both the AB and C regions?

The reviewer raises a very interesting point. To our knowledge all described variants of Hsf1 that do not trimerize anymore have a deleted HR-A and/or HR-B (Zuo et al. 1994) and as such would not be useful for the suggested experiment. Based on published data on Hsf1-deletion mutants (Zuo et al. 1994) and our HX-MS results showing strong protection in HR-B, suggesting interaction of HR-C with HR-B, we tried to construct a suitable mutant by exchanging hydrophobic residues of the heptad repeat region A, which was proposed to be responsible for the formation of a trimeric coiled-coil and was shown recently to form the trimer interface, by serine. In HX-MS experiments both regions, HR-A and HR-C, were completely exchanged after 10 min pre-incubation at all temperatures tested, indicating that the mutated residues are necessary for the interaction of HR-A with HR-C and for trimerization.

The effect of Hsp90 on the HDX reactions (Figure 6A and B) has been carried out on the intact Hsf1. The apparent cooperativity between the effect of temperature on HDX by HR-AB and H-C limits the ability of these experiments to discriminate between the mechanisms proposed in Figure 7. But mutagenesis of Hsf1 has the potential to isolate the effects of Hsp90 to interactions with one domain or the other. Has this been tried?

Unfortunately, our data do not provide any indication where Hsp90 interacts with Hsf1. This might be due to an incomplete sequence coverage in the presence of the excess of Hsp90, which leads to overloading certain regions of the mass spectra preventing evaluation of a number of Hsf1 derived peptides. In the absence of any information on the interacting domain, mutagenesis would be pure guesswork and has not been undertaken so far.

*Following melting of the AB-C (intramolecular) complex, oligomerisation competes with complex re-formation. Hence the concentration dependence of the relationship between Hsf1 concentration and the thermal sensing profile. Is it possible to model this relationship to predict the differences in Hsf1 needed to explain tissue-specific (testis versus other tissues) and heterologous cell type expression effects on temperature sensing? Are the predicted differences in Hsf1 concentration reasonable?*

Following the suggestion of the reviewer we tried to get an estimate of the heat shock induction temperatures in cells and added the following to our Discussion section:

Our dimer activation model assumes that the proposed Hsf1 dimer has a lower T_m_ (T_m,D_) than the Hsf1 monomer and that the measured T_m_ depends on the fraction of Hsf1 dimer present in the assay. […] This is well within the range that would lead to a functional heat shock response according to our model.

*Reviewer #2: Overall, the data are convincing and highly intriguing. The idea that the HR-C motif is crucial to HSF-1 trimerization and DNA binding (via intramolecular interactions with the HR-A/B domain) is not novel as also very correctly cited by the authors themselves. But, especially for the human HSF-1, the finding that it is the temperature sensing domain was never directly demonstrated before. The finding that Hsp90 is not inhibiting trimerization is the most surprising finding and challenges a long-lived dogma in the field. As stated again correctly by the authors themselves, it cannot be excluded that in vivo co-chaperones may still play a role, but this will have now to be shown in separate studies. My question/comments that the authors may wish to consider are: 1) Whereas the data clearly demonstrate that Hsp90 (alone) does not negatively regulate HSF-1, I am somewhat puzzled the fin dings that HSP90 lowers the threshold temperature for HR-C unfolding. Also in both models presented in Figure 7, this does not become clear. Whilst in the dimer activation model, the effects of HSP90 on HR-A/B might be explained by assuming stabilization of the intermediate dimer, I do not understand how such would explain effects on HR-C unfolding. I further would like to add that I find Figure 7 and the related explanation (Discussion, fourth paragraph) not very clear in general.*

We have restructured and rewritten this part to make it clearer. We also included additional information based on published data and the recent crystal structure of the trimerization domain of *Chaetomium thermophilum* Skn7, supporting our novel model of HSF1 activation.

The principal idea behind this model is that the interaction of HR-C and HR-A/B is stronger in the monomer than in the dimer. The stability of HR-C as a single helix depends crucially on interaction with HR-A/B. The stronger the interaction of HR-C with HR-A/B the more stable HR-C. Thus, weakening the interaction of HR-C with HR-A/B will destabilize HR-C and lower the energy required to unfold HR-C. We included the following sentences in the revised manuscript:

“Hsp90 could modulate the monomer-trimer transition by stabilizing HR-A-HR-A interactions and/or destabilize HR-A/B-HR-C interactions, resulting in a reduced dimer dissociation rate and an increased rate of trimerization at lower temperatures. Stabilization of the HR-A-HR-A dimer and concomitant destabilization of HR-A/B-HR-C interaction would automatically destabilize HR-C since single helices are not stabile in solution and only stabilized by interaction with other structural elements, leading to a reduced unfolding transition temperature.”

*2) Whilst the data strongly suggest HSF-1 as thermosensor and thus the main driver activation of the heat shock response at elevated temperature, HSF-1 can also be activated at physiological temperatures by various forms of proteotoxic stress. The existing model of negative regulation by Hsp90 or TRiC explained this by a shift of the chaperones from HSF-1 binding to binding to the stress-unfolded proteins. Can the authors speculate how they would interpret that data with their model and findings that Hsp90 does not negatively regulate HSF-1 activation?*

The reviewer touches a very interesting point. We have included the following paragraph into our Discussion section:

“How could Hsf1 be active at non-heat stress conditions, for example during development (Xiao et al., 1999), and how could it be activated by salicylate, low pH, Ca^2+^-ions, hypoxia, or proteotoxic stress other than heat shock as has been demonstrated previously (Mosser et al., 1990; Jurivich et al., 1992; Huang et al., 1995; Liu et al., 1996, Zhong et al., 1999; Ahn and Thiele, 2003)? […] However, Hsf1 dimerization could also be affected directly by changing local concentration, as through transport of Hsf1 into the nucleus (Dai et al., 2003) or preventing its export (Vujanac et al., 2005), and by posttranslational modification, including glutathionylation of the cysteine in HR-A in response to oxidative stress or alkylating agents (Liu et al., 1996), and phosphorylation of Thr142 in HR-A (Soncin et al., 2003), both of which would reduce the positive net charge of HR-A and thus the electrostatic repulsion.”

Reviewer #3: This manuscript addresses an important aspect of the regulation of the heat shock repsonse. In eukaryotic cells, the trimerization of the transcription factor Hsf1 is important for its activation. This process is affected by the molecular chaperones Hsp70 and Hsp90. The authors make excellent use of H/D exchange technology to obtain new insight on the underlying temperature-induced processes which finally lead to trimerization, including the influence of phosphorylation of Hsf1 and Hsp90 on the transition temperature of this process. Some technical issues need to be addressed. Specific points:

*1) Most of the experiments are based on H/D exchange. They reveal changes in local dynamics and accessibility depending on temperature variation. It is reasonable to assume that the defined changes identified by the authors are the critical changes that promote dimerization. However, as a proof of principle one would like to see an assay that reports directly on quaternary structure performed in parallel to some of the H/D experiments. This may also provide interesting information on the coordination of these events.*

We have performed the requested control experiments analyzing Hsf1 after pre-incubation at different temperatures by blue native gel electrophoresis with similar results (new Figure 2B and Figure 2—figure supplement 1). Please also see above, major point 1.

*2) The authors show in Figure 1C that monomeric Hsf1 forms trimers upon incubation at 42 degrees for 10 min, but did the authors also check what happens in the temperature range they used for HDX i.e. 30-45 degrees (the temperature region where a decrease in HX in HR-A/B is observed as shown in Figure 2D)?*

This has been done (new Figure 2B and Figure 2—figure supplement 1).

*3) In Figure 2B no significant changes are observed in deuterium incorporation in the DBD at all the temperatures tested. I expected to see some changes in the DBD because trimerization of Hsf1 induces DNA binding and hence should cause certain conformational changes in the DBD to enable it to bind to DNA.*

A conformational change in the DNA binding domain is not necessary. The isolated DNA binding domain is also able to bind to DNA, albeit with low affinity. According to Kim et al. 1994 the K_D_ for binding of the isolated DNA binding domain of *Drosophila* Hsf1 to the cognate motif nGAAn is 2.2 µM at physiological salt concentrations (150 mM KCl) and 4°C and is actually increasing with increasing temperature (up to 18°C tested). After trimerization the K_D_ for binding to the consensus heat shock element (nTTCnnGAAnnTTCn) is expected to be much lower (in our experiments the K_D_ is 1 nM) due to the increased avidity.

Of note, intrinsic hydrogen exchange rates are of course higher at higher temperatures and proteins tend to exchange protons more rapidly at higher temperatures due to increased conformational dynamics. However, all incubations in D_2_O were performed at 20°C. Since we did not observe significant changes in protection in the DNA binding domain, conformational changes in the DNA binding domain potentially induced by the pre-incubation at elevated temperatures seem to be completely reversible in contrast to the changes induced in HR-A/B and HR-C.

4) In fluorescence anisotropy experiments, the authors fitted all curves to the same equation assuming identical K_D_ values. However, for example, at 30 and 37/39/42 degrees, the shape of the red curve (5000 nM) appears different. At 30 degrees it is exponential while at 37/39/42 degrees, it is sigmoidal.

*Moreover, Hsf1 was incubated at different concentrations at different temperatures to enable trimer formation and later serially diluted. Did authors check whether the initial trimerization state of Hsf1 obtained during incubation at different temperatures is maintained upon serial dilutions?*

We did this control experiment, displayed in new Figure 3G.

*Error bars should be shown in Figure 4.*

We added the error bars to previous Figure 4 now Figure 6.

*5) In a Figure 1C, band is obtained at a size larger than the trimer. Does that mean that higher ordered oligomers are formed instead of trimers?*

As mentioned in the text, higher order oligomers are formed in addition to trimers. However, upon dilution this band disappears suggesting that the higher order oligomers dissociate (see Figure 3G).

*6) The authors fitted the temperature response curves in the HDX experiments to the thermal equilibrium unfolding two state equation. An important parameter for the equilibrium unfolding fit is the reversibility of the reaction. Did authors check whether Hsf1 thermal unfolding is reversible?*

We did check for reversibility and had already stated in the original manuscript that trimerization was not reversible in our hands. We have now added more quantitative data on this. We heat shocked Hsf1 for 10 min at 42°C, then incubated the sample for various time intervals at 20°C, before analyzing the conformation of Hsf1 by HX-MS (included as new Figure 3A–F). We also state this clearly in the manuscript. We used the unfolding fits only for determining the T_m_ for the given conditions. We do not derive the unfolding enthalpy or heat capacity of the protein. This is now stated more clearly in the manuscript. The equilibrium unfolding equation fits the data reasonably well with residuals generally smaller than the deviations between biological replicates and no systematic deviation detectable. Therefore, we decided to use this equation instead of a generic three-parameter sigmoidal equation.

*7) H/D exchange is temperature-dependent: the higher the temperature, the faster the exchange. In the experiments, Hsf1 was incubated at different temperatures and then diluted 1:20 at 20 degrees. The authors may want to add control experiments in which they show whether pre-incubation influences the exchange.*

I am not quite sure what the reviewer wants to point out with this comment. The purpose of this study was to detect conformational change induced by pre-incubation of Hsf1 at elevated temperatures. To exclude the effect of temperature on the intrinsic exchange kinetics as well as on the dynamics of the protein all HX-experiments were performed at 20°C. In preceding experiments we have also performed HX with Hsf1 at higher temperatures and observed the increase in exchange kinetics expected. Now we also added the control experiment to show that the induced conformational changes do not revert back during an incubation at 20°C for 3 to 100 min (Figure 3).

8) MS/MS spectra of the two peptides AA[159-168] and AA[389-395] discussed intensively could be added to a supplement. Furthermore, it may be useful to show difference plots for the effect of Hsp90 (similar to Figure 2B) at two different temperatures.

We now show MS/MS spectra for the most important peptides (Figure 2—figure supplement 4) and we added the difference blot of deuteron incorporation into Hsf1 in the presence of Hsp90 minus the deuteron incorporation of Hsf1 in the absence of Hsp90 (new Figure 8A) for all temperatures measured.

*Reviewer #4: The authors provide a potentially very interesting manuscript describing how Heat Shock Factor 1 might serve as a thermosensor for transducing a heat-stress signal in cells along with a role for the Hsp90 molecular chaperone in broadening the temperature response range of HSF1. They use an elegant biophysical approach (hydrogen-deuterium exchange coupled with mass spectrometry (HX-MS)) to delineate the polypeptide areas of HSF1 that are sensitive to temperature changes. Their work identifies a region that unfolds with temperature (HSF1 regulatory region) and an area that concomitantly tightens (trimerization region). The relative structural transitions in these regions in response to temperature are relatively sharp especially compared to the behavior in the presence of Hsp90, which broadens the structural transitions. In addition, the authors present data suggesting phosphorylation might also impact the responsiveness of HSF1 to temperature. In considering the work for publication the following points should be weighed: 1) Isolating monomeric, activatable HSF1 is challenging. The authors argue they have accomplished this feat. As this reagent is key to all of the presented findings it is imperative that is well characterized.*

*The basic model is that monomeric HSF1 will not bind DNA but heat stress will induce trimerization and a DNA binding activity. Yet, in Figure 1D there is clear DNA binding by what is supposed to be monomeric HSF1 since in lane 2 it has not been exposed to elevated temperature. Is this DNA binding resulting from spontaneous trimerization of their HSF1 or is monomeric HSF1 binding DNA?*

The reviewer is correct, there is some DNA binding observed in the monomer preparation of Hsf1. We discovered that freezing and thawing leads to spontaneous trimerization of monomeric Hsf1. We therefore used in many experiments HSE-DNA coupled to magnetic beats to fish out any trimerized Hsf1. In all HX-MS experiments the amount of trimeric Hsf1 was too small to be detected as long as Hsf1 was incubated on ice.

*The authors should determine if HSF1 is trimerizing under their control conditions by incorporating a crosslinker and then probing for trimeric species of HSF1 by western blot analysis.*

Instead of using a crosslinking assay, which is not quantitative, we used blue native gel electrophoresis to determine trimeric and monomeric DNA and also to demonstrate that incubation at elevated temperature increases the amount of trimeric Hsf1 now shown in Figure 2B. Also, Figure 3G lane 8 shows that our Hsf1 preparation used for experiments contains practically no detectable trimeric Hsf1.

*The presence of some trimeric HSF1 under control conditions is reasonable; however, the relative levels need to be determined and the presence of this species in their HX-MS studies needs to be considered.*

As mentioned the amount of trimeric Hsf1 in HX-MS experiment was so low that it had no impact on the determination of the temperature response curves. Assuming that the fraction of low exchanging species detected for peptides 159-168 and 169-175 of region HR-A/B detected at 20°C corresponds to the amount of trimeric Hsf1 present in the preparation then its concentration was less than 10% (Figure 2E). Similarly, the amount of high exchanging species for peptides of region HR-C, potentially corresponding to present trimer, was below 10% (Figure 2G).

2) While the authors present the baseline HX-MS data for control HSF1, the baseline data for fully activated HSF1 should also be given (i.e., the experimental results use 10 min exposures to the various temperatures but what are the HX-MS data for recombinant HSF1 exposed for prolonged times). Showing HX-MS data from equilibrated trimeric HSF1 will help in assessing the presenting kinetic activation data (Figure 3).

We now provide HX-MS data with prolonged pre-incubation of Hsf1 at the different temperatures (included in Figure 2E and G; and Figure 2—figure supplement 3).

*In addition, the kinetic data requires validation studies to show that the changes they observe in HX correlate with acquisition of HSF1 trimers and DNA binding activity, which could be accomplished by including crosslinking and EMSA analysis of HSF1 exposed to similar conditions.*

The reviewer might have overlooked it, but in previous Figure 6C (now Figure 8C) we show an EMSA analysis of the temperature dependent activation of Hsf1 in absence and presence of Hsp90, which were performed exactly as the HX-MS experiments in other parts of the study, yielding within experimental error identical results. We also present now a temperature transition curve from blue native gel electrophoretic analysis of the quaternary state of Hsf1 (new Figure 2B and new Figure 2—figure supplement 1).

*3) The authors present a potentially interesting concept that HSF1 concentration might influence its temperature activation properties (Figure 4). Given the novelty of this concept, in vivo data correlating with the in vitro work should be presented. For example, is HSF1 much more abundant in testis where its activation temperature is lower relative to other tissue types? Do all tissues with high levels of HSF1 display lower HSF1 activation responses? In addition, how do the shown concentrations of HSF1 compare to in vivo levels in any tissue type under normal or disease states? Changes in HSF1 concentrations in cancer cells would be especially interesting in light of the recent reports from the Lindquist laboratory showing constitutively active HSF1 in cancer cells.*

As the reviewer states, the physiological relevance of our findings needs to be investigated. We have carefully screened the literature. The concentration of Hsf1 in testis has not been determined nor in any other tissue. The only values that we could find are a relative quantification of the complete proteome of 11 cancer cell lines performed by mass spectrometry in the Mann laboratory (Geiger et al. 2012). From these values we were able to calculate the fraction that Hsf1 makes up relative to all proteins detected. However, the value for cellular protein concentration is not trivial to determine, because for this one needs the volume of the cells. In many reviews values of 200 to 300 mg/ml are given, most of the time without any reference and if a reference is given, then it is another review without reference. We screened the literature extensively and found for HEK293 and Jurkat cells a protein concentration of 154 and 127 mg/ml (Gillen and Forbush 1999; Fumarolo et al. 2005). In the revised manuscript we include now some estimations of Hsf1 concentration in mammalian cells based on these values. However, Hsf1 is not evenly distributed in the cells but shuttles in and out of the nucleus (Vujanec et al. 2005). It becomes clear that what this reviewer asks is not trivial and requires a careful study to come to trustworthy conclusions. We will try to address this question in the future.

*4) The data generated with the phosphomimic mutants in Figure 5B are interesting. However, given the sensitivity of HX-MS it is quite possible that the amino acid substitutions are causing pleotropic effects. Hence, this work would require an assessment of actual phosphorylated HSF1 in order to be substantiated.*

This reviewer certainly has a valid point that phosphomimetic amino acid substitutions may not reflect the real situation. She/he is probable also aware of the fact that protein phosphorylation in vitro is usually performed using radiolabeled [γ-^32^P]ATP because the efficiency of in vitro phosphorylation is not very high, to say the least. For HX-MS experiments we would need phosphorylation levels of >90%. This is not achievable.

*5) The work with Hsp90 is also intriguing. However, is this a general chaperone effect or is it specific to Hsp90? Would Hsp70 have a compare impact on the HX-MS data?*

It would certainly be interesting to compare the influence of Hsp90 on Hsf1 with the influence of Hsp70. However, this is not as trivial as the reviewer might think and involves a completely new study. First, addition of another protein in HX-MS experiments requires a new establishment of the analysis. Since the chaperone is added in several fold excess and, depending on the digestion efficiency of the chaperone, peptides will overlap with Hsf1 peptides, the liquid chromatography gradient has to be adjusted to be able to separate the relevant peptides. Second, Hsp70 cannot be used alone but should be used in combination with one of the some 16 J-domain co-chaperones predicted to be present in the cytosol of human cells and maybe one of the seven-nucleotide exchange factors. At present we have no idea which of the J-domain protein and which of the nucleotide exchange factor is relevant for Hsf1 regulation. Also, should the heat inducible Hsp70 or the constitutive Hsc70 be used in such a study. This is a completely new study. Besides, Hsp70 was shown not to affect activation of Hsf1 but attenuation (Rabindran et al., 1994 Mol Cell. Biol).

*While the following is a bit outside the scope of this work, if we assume Hsp70 does impact HSF1 and given the authors focus on identifying factors that influence the HSF1 temperature set point, would the testis-specific Hsp70 have a distinct impact on HSF1 structural transitions relative to the more broadly expressed Hsp70s?*

This would certainly be an interesting new study.
